# Directed evolution of rRNA improves translation kinetics and recombinant protein yield

**DOI:** 10.1038/s41467-021-25852-5

**Published:** 2021-09-24

**Authors:** Fan Liu, Siniša Bratulić, Alan Costello, Teemu P. Miettinen, Ahmed H. Badran

**Affiliations:** 1 The Broad Institute of MIT & Harvard University, Cambridge, MA 02142 USA; 2 Department of Chemistry, The Scripps Research Institute, La Jolla, CA 92037 USA; 3 Koch Institute for Integrative Cancer Research, Massachusetts Institute of Technology, Cambridge, MA 02142 USA; 4 Present Address: Department of Biology and Biological Engineering, Chalmers University of Technology, 412 96 Göteborg, Sweden

**Keywords:** Synthetic biology, Catalytic RNA, Ribosome

## Abstract

In bacteria, ribosome kinetics are considered rate-limiting for protein synthesis and cell growth. Enhanced ribosome kinetics may augment bacterial growth and biomanufacturing through improvements to overall protein yield, but whether this can be achieved by ribosome-specific modifications remains unknown. Here, we evolve 16S ribosomal RNAs (rRNAs) from *Escherichia coli*, *Pseudomonas aeruginosa*, and *Vibrio cholerae* towards enhanced protein synthesis rates. We find that rRNA sequence origin significantly impacted evolutionary trajectory and generated rRNA mutants with augmented protein synthesis rates in both natural and engineered contexts, including the incorporation of noncanonical amino acids. Moreover, discovered consensus mutations can be ported onto phylogenetically divergent rRNAs, imparting improved translational activities. Finally, we show that increased translation rates in vivo coincide with only moderately reduced translational fidelity, but do not enhance bacterial population growth. Together, these findings provide a versatile platform for development of unnatural ribosomal functions in vivo.

## Abstract (web summary)

Ribosome kinetics are rate-limiting for protein synthesis. Here the authors evolve diverse 16S rRNAs for enhanced protein synthesis rates and genetic code expansion efficiencies in vivo.

## Introduction

Directed evolution of ribosomal rRNA (rRNAs)^1,2^ towards unnatural bioactivities has highlighted plasticity in the cellular translation apparatus. Mutations to rRNAs or ribosomal proteins (r-proteins) can enhance translational fidelity^3^, reduce translational kinetics^4^, endow antibiotic resistance^5^, affect ribosome assembly^6^, and enhance the incorporation of noncanonical monomers or decode nonsense codons^1^. However, it remains unknown whether the translation rate or catalytic potential can be increased by ribosome-specific modifications.

Given the large sequence space of an rRNA, powerful methods of directed evolution are needed to access improved kinetic translation capabilities. Classical directed evolution requires extensive effort to maximize library diversity, fine-tune sequential selection conditions, and suffers from limitations in their mutational spectrum and library transformation efficiencies^7^.

A high-throughput methodology for directed evolution of rRNAs could therefore facilitate unbiased investigations of ribosomal translation and may enable researcher-dictated biosynthetic capabilities. In particular, orthogonal translation systems, which create dedicated pools of researcher-controlled ribosomes that are decoupled from cellular viability^8^, have enabled the exploration of sequence-function relationships en route to unnatural bioactivities^1^, permitted investigations into a mutation of sequence essential for cell function, and enabled the discovery of augmented ribosomal activities^9^.

Bolstered by this decoupled translation framework, we developed an orthogonal ribosome-dependent phage-assisted continuous evolution (oRibo-PACE) methodology that enables rapid directed evolution of rRNAs towards researcher-defined activities. We use this system to explore the interplay between translational kinetics and fidelity through the evolution of 16S rRNAs from three bacterial species. We characterize evolved rRNA mutants through variable reporter gene and context dependencies in an orthogonal translation system, and find two of three starting rRNA scaffolds evolved variants that achieve higher kinetic translation rates than those of wild-type *E. coli* rRNA in an *E. coli* host. The discovered variants function in a context-independent manner when evaluated using variable reporter gene, ribosome-binding site (RBS), and alongside cognate heterologous r-proteins. Through analysis of the evolved mutations, we identify consensus mutations that impart improved translational activities to a wide repertoire of heterologous ribosomes. Critically, evolved rRNAs furnish ribosomes capable of greatly increasing the yield of proteins bearing noncanonical amino acids in an orthogonal translation system. Finally, we extend these findings to generate cells harboring only evolved rRNA variants, showcasing elevated proteome-wide translation rates as compared to wild-type *E. coli* rRNA, with only minor reductions in translational fidelity. Our findings showcase that ribosomes can be evolved for improved protein yield, enhanced genetic code expansion, and faster translation rates in living cells.

## Results

### Development of a PACE-compatible orthogonal translation system

**Fig. 1:**
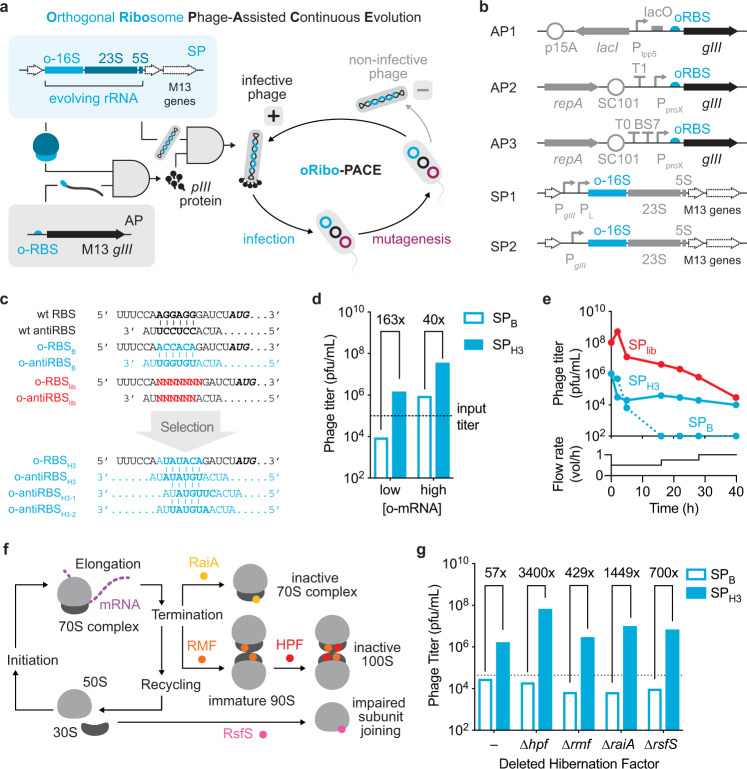
Development of a PACE-compatible selection for orthogonal translation. **a** Schematic representation of an orthogonal rRNA-dependent PACE selection. An engineered M13 bacteriophage (selection phage; SP) encodes the o-rRNA operon in place of *gIII*. Upon infection, functional orthogonal ribosomes efficiently translate *gIII* from the accessory plasmid (AP), yielding infectious phage progeny. Efficient o-rRNA diversification is implemented via a mutagenesis plasmid (MP). **b** AP and SP designs used in directed evolution campaigns. **c** A comparison of native and orthogonal RBS/antiRBS pairs used in this study. **d** Preliminary analysis of o-rRNA-dependent phage production under low (0 mM IPTG) or high (1 mM IPTG) mRNA concentrations using AP1_H3_ (*n* = 2 biological replicates). **e** Discovery of o-antiRBS variants under continuous culturing conditions using a degenerate library in the SP-borne o-rRNA. **f** Schematic representation of known ribosome hibernation factors. **g** Comparison of phage enrichment assays using the constitutive AP2_H3_ (top) in wild-type host (S2060) or host cells where ribosome hibernation factors have been deleted: hibernation promoting factor (∆*hpf*), ribosome modulation factor (∆*rmf*), ribosome-associated inhibitor A (∆*raiA*), or ribosomal silencing factor S (∆*rsfS*) (*n* = 1 biological replicate). Source data are available in the Source Data File.

PACE has facilitated the exploration of sequence-function relationships of biomolecules with diverse cellular activities^10–16^. Briefly, PACE exploits the rapid M13 bacteriophage lifecycle and couples the production of plasmid-borne *gIII*, encoding the minor coat protein pIII necessary for both bacterial infection and membrane extrusion^17^, to the activity of the evolving biomolecule encoded on a pIII-deficient phage genome. The genetic diversity of the evolving biomolecule is easily tuned through a small molecule-inducible expression of mutator proteins from the mutagenesis plasmid (MP)^18^. Historically PACE has been limited to protein-coding genes. We envisioned that PACE could be extended to the directed evolution of orthogonal rRNAs (o-rRNAs), allowing efficient traversal of mutational landscapes and uncovering variants with altered translational activity (Fig. 1a). To establish an o-rRNA PACE selection in *E. coli*, we adapted an orthogonal translation genetic circuit^19,20^ to integrate the M13 bacteriophage *gIII* (which encodes pIII), yielding the Accessory Plasmid 1 architecture (AP1; Fig. 1b) and concurrently engineered selection phages (SPs) to encode the complementary o-rRNA operon. Functional o-rRNAs capable of forming active ribosomes and translating the *gIII* mRNA using the o-RBS would robustly produce pIII, yielding infectious phage progeny.

While the previously reported o-antiRBS_B_ efficiently directs translation of an sfGFP reporter bearing the cognate o-RBS_B_ sequence (Fig. 1c)^8^, a direct adaptation of o-RBS_B_ to AP1 rendered S2060^13^ cells uninfectable by wild-type M13 phage, indicating high background pIII expression. Thus, we developed a two-stage selection to identify PACE-compatible o-RBS/o-antiRBS pairs with reduced background translation by host ribosomes. We first introduced a degenerate library of 4^7^ RBS variants (o-RBS_lib_, Fig. 1c) and assessed the infectivity of the resultant cells to identify sequences poorly recognized by host ribosomes (Supplementary Fig. 1a). This analysis revealed 33 putative o-RBS candidates, and we further characterized the most abundant seven variants (Supplementary Fig. 1b). To discover potential cognate o-antiRBSs, we introduced a degenerate library of 4^6^ antiRBS variants in the SP-borne *E. coli* o-rRNA (o-antiRBS_lib_, Fig. 1c) into *E. coli* host cells bearing each of the seven o-RBSs. Functional o-antiRBS sequences should efficiently translate *gIII* and give rise to progeny phage (Supplementary Fig. 1c–e). After further optimization of spacer sequences (Supplementary Fig. 1f), we identified o-RBS_H3_ (Fig. 1c) as the optimal orthogonal sequence for subsequent experiments. We adapted o-RBS_H3_ to AP1 (AP1_H3_) yielding 40- to 163-fold enrichment for SPs encoding the cognate o-antiRBS_H3_ (SP_H3_) relative to SPs bearing the mismatched o-antiRBS_B_ sequence (SP_B_) (Fig. 1d).

While the o-RBS_H3_/o-antiRBS_H3_ pair enabled phage propagation in standing culture (Fig. 1d), we hypothesized that alternative solutions may exist under continuous culture conditions. We continuously propagated a degenerate SP library encoding 4^6^ antiRBSs (o-antiRBS_lib_, Fig. 1c) using AP1_H3_ in S2060 cells yielding comparable phage titers to SP_H3_, while SP_B_ was rapidly washed out (Fig. 1e). We analyzed the resulting SP populations at 40 h by Sanger sequencing (24 clones) and found that SP_lib_ converged on exclusively two variants: o-antiRBS_H3-1_ and o-antiRBS_H3-2_ (Fig. 1c). Both variants robustly translate a LuxAB luciferase reporter, showing a similar dynamic range to the initial o-antiRBS_H3_ variant (Supplementary Fig. 1g). We note that o-antiRBS_H3-1_, but not o-antiRBS_H3-2_, appeared in our initial antiRBS library (Supplementary Fig. 1e), suggesting differential o-ribosome activities may depend on culturing conditions.

Although we successfully identified functional o-antiRBS sequences from an unbiased SP library, the final phage titers were considerably lower than those in previous protein-based PACE campaigns^10,11,13–16^. We noted that host cells in the turbidostat reside at the transition between the exponential and stationary phase, during which o-rRNAs may be inactivated by hibernation factors^21^. Accordingly, we deleted factors known to inhibit ribosome activity to improve the propagation of o-rRNA SPs (Fig. 1f). Deletion of ribosome hibernation promoting factor (HPF) from S2060^13^ yielded host strain S3317, with a 3400-fold improvement in SP propagation (Fig. 1g). Concurrently, we prepared an AP architecture, AP2 (Fig. 1b), which encoded a growth phase-independent constitutive promoter^22^ to simplify o-rRNA evolution experiments (Supplementary Fig. 2a–d) and integrated the pSC101 origin of replication for stringent copy number control^23^. When introduced into S3317 cells, AP2_H3_ supported SP_H3_ propagation 4831-fold more efficiently than the mismatched SP_B_ (Fig. 1g and Supplementary Fig. 2e).

We next competed all o-antiRBS SPs (Fig. 1b) using S3317/AP2_H3_ under continuous flow at varying lagoon dilution rates. We note that low lagoon flow rates (<1.0 vol h^−1^) led to poor SP propagation, consistent with ribosome inactivation at saturated cell densities (Supplementary Fig. 2f)^21^. We analyzed individual SPs propagated at 2 vol h^−1^ using Sanger sequencing and found that most SPs encoded o-antiRBS_H3-1_, in agreement with the SP_lib_ evolution experiment (Fig. 1e and Supplementary Fig. 1e). In overnight enrichment assays in standing culture, SP_H3-1_ similarly showed improved titers (up to 186-fold) over SP_H3_ (Supplementary Fig. 2g). Following additional strain engineering (∆*fhuA*^24^) to produce S3489 (Supplementary Fig. 2g, h) and plasmid modification to yield the AP3 architecture (Fig. 1b) to limit AP/SP recombination (Supplementary Fig. 2b, i–l), we found the S3489/AP3_H3_/SP_H3-1_ combination to be the optimal orthogonal translation system and used it for all subsequent experiments.

### Continuous directed evolution of orthogonal ribosomes

**Fig. 2:**
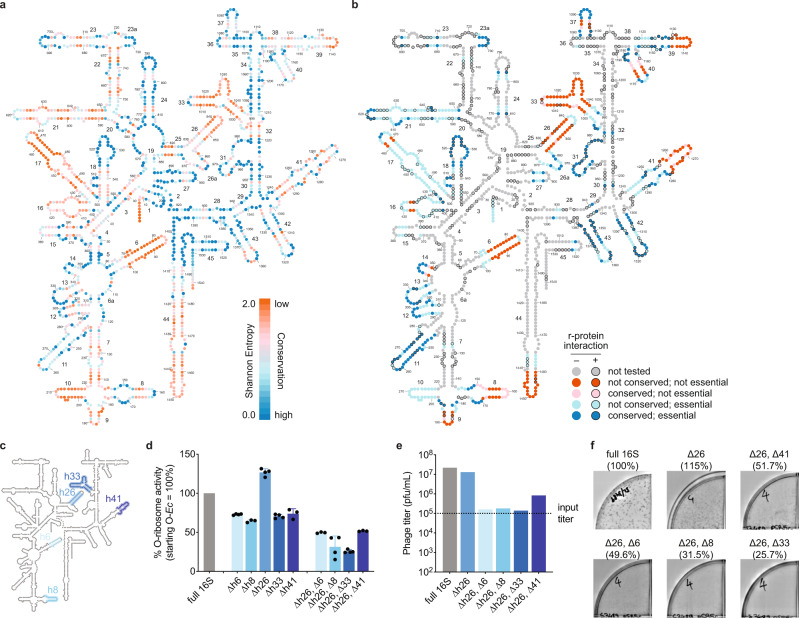
Establishing EP–SP correspondence via *E. coli* 16S rRNA truncation analysis. **a** Nucleotide conservation of the 16S rRNA which was used to guide truncated rRNA studies. Structure generated via Ribovision^60^. **b** Composite of tested 16S rRNA truncations and binned by their effects on orthogonal sfGFP reporter translation. Variants with sfGFP output below 25% are considered inactive. **c** Key deletions used in the SP analysis as mapped on the *E. coli* 16S rRNA secondary structure. **d** Single and double 16S rRNA truncations variably affect orthogonal GFP reporter translation, providing a gradient of activities for SP-based analyses. Data are normalized to untruncated *E. coli* 16S o-rRNA. Data reflect the mean and standard deviation of 3–4 biological replicates (*n* = 3-4). **e** Enrichment assays of SPs encoding full-length and truncated *E. coli* 16S o-rRNAs. **f** Plaque assays showing the relationship between 16S o-rRNA activity and plaque formation. Labels indicate the truncation and activity in orthogonal GFP reporter translation relative to the untruncated 16S *E. coli* o-rRNA. Data reflect the mean and standard deviation of 1–11 biological replicates (*n* = 1–11). Source data are available in the Source Data File.

We and others have recently shown that rRNAs derived from heterologous microbes can robustly support *E. coli* viability upon deletion of all host-derived rRNAs^20,25^. As only *E. coli*-derived o-rRNAs have been successfully evolved to date^1,26^, we hypothesized that diverse heterologous o-rRNA sequences may undergo distinct evolutionary trajectories in PACE, yielding variable solutions to identical selection conditions. However, divergent heterologous ribosomes often suffer from reduced starting activity in an *E. coli* chassis as compared to wild-type *E. coli* ribosomes^20^. The 16S rRNA is a highly conserved sequence, yet encoding poorly conserved residues often residing at the 3-dimensional periphery of the ribosome (Fig. 2a). To define a threshold for heterologous ribosome activity, we generated deletions in *E. coli*-derived 16S o-rRNA and characterized their activity levels using reporter and SP enrichment assays (Fig. 2b–f). These experiments established that SPs bearing o-rRNAs with activity levels ≥32% of WT *E. coli* o-rRNA robustly propagate under stringent conditions.

**Fig. 3:**
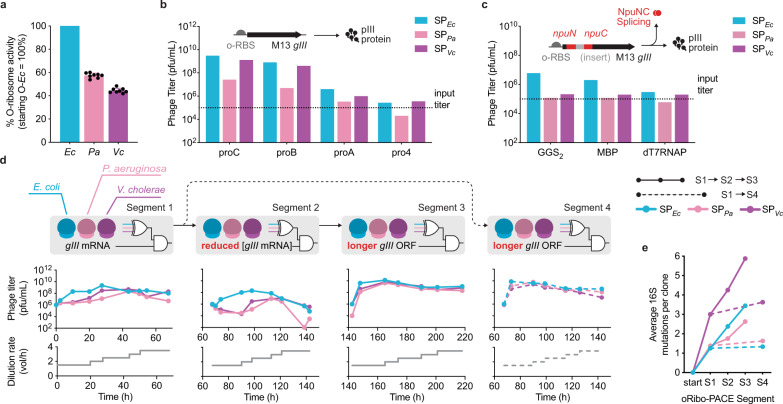
Continuous directed evolution of orthogonal ribosomes. **a** Starting o-ribosome activity of *E. coli* (*Ec*), *P. aeruginosa* (*Pa*), and *V. cholerae* (*Vc*) o-rRNAs, quantified using sfGFP production. Data reflect the mean and standard deviation of 8 biological replicates (*n* = 8). **b** Phage enrichment assays of SP_Ec_, SP_Pa_, and SP_Vc_ in S3489 cells using APs encoding promoters of decreasing strength. **c** Phage enrichment assays of SP_Ec_, SP_Pa_, and SP_Vc_ in S3489 cells encoding variable inserts within the ^intein-proB^AP_H3_ architecture: GGS2 linker, MBP, and dT7RNAP. **d** Summary of PACE evolution trajectories. In the first trajectory, oRibo-PACE was carried out in three segments (segments 1 → 2 → 3). In the second trajectory, a shorter oRibo-PACE campaign was carried out in two segments (segments 1 → 4). In all segments, high levels of mutagenesis (MP6)^18^ were induced. Phage titers sampled during o-ribosome evolutions and lagoon flowrates are shown on the bottom. **e** The average number of mutations per sequenced clone is highest in SP-borne o-rRNA derived from *V. cholerae*, followed by that of *E. coli*, while o-ribosome from *P. aeruginosa* on average had the lowest number of mutations at the end of each PACE segment. Colors blue (*E. coli*), pink (*P. aeruginosa*), and purple (*V. cholerae*) are consistent across plots. Source data are available in the Source Data File.

Next, we identified *P. aeruginosa* (Pa) and *V. cholerae* (Vc) heterologous o-rRNAs as promising candidates for oRibo-PACE, as they showed comparable activity to *E. coli*-derived o-rRNA (Fig. 3a) and could successfully propagate in standing culture, albeit at lower efficiency than their *E. coli* counterpart (Fig. 3b). To evolve heterologous rRNAs, we subjected starting rRNA species to multi-stage selection regimes with increasing selective pressure. We performed 218 h (~268 generations^10^) of PACE using *E. coli* (SP_Ec_), *P. aeruginosa* (SP_Pa_), and *V. cholerae* (SP_Vc_) o-rRNAs while varying selection stringency over multiple segments (Fig. 3b–d). In all segments, we employed a previously optimized MP, MP6^18^, to enhance o-rRNA sequence diversity, and regularly increased lagoon flowrates to enhance selection stringency.

In the first segment (S1 = 0–68 h), we diversified the clonal SP-borne o-rRNAs through genetic drift by employing a constitutive promoter driving *gIII* expression from AP3_H3_ (proB^22^; Fig. 3b, d). During the second segment (S2 = 68–143 h), we increased selection stringency by reducing the *gIII* promoter strength 8-fold ((pro4^22^); Fig. 3b, d), resulting in a > 250-fold decrease in SP propagation efficiency (Fig. 3b). During the third segment (S3 = 143–218 h), we incorporated a split-intein pIII^14^ strategy where an inserted protein sequence increased the effective length of *gIII* by 123% (425–947 amino acids) and decreased SP propagation efficiency further by >120-fold (Fig. 3c, d and Supplementary Fig. 3a–g). Finally, to examine the effect of selection schedule on o-ribosome variant activities, we carried out a fourth segment (S4 = 68–143 h) using the split-intein pIII approach and SP populations immediately following genetic drift (S1→S4) to compare a shorter selection regime to the aforementioned longer version (S1→S2→S3) (Fig. 3d). We note that all SP populations robustly propagated across all segments, with the exception of SP_Pa_ during S2 (Fig. 3d and Supplementary Fig. 3f), which rebounded during subsequent high stringency selection, suggesting accumulation of mutations to enable enhanced o-rRNA activities. Furthermore, all three SP populations underwent cognate 23S rRNA deletion at virtually identical time points during oRibo-PACE (Supplementary Fig. 3h), reflecting complementation with the host *E. coli* 23S rRNA as previously described^20^.

**Fig. 4:**
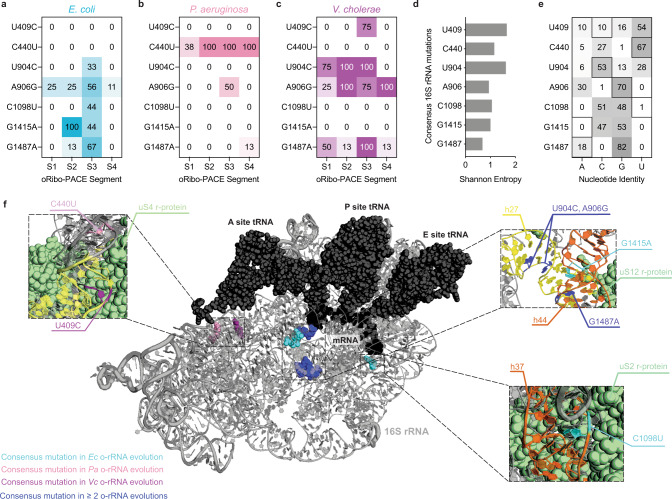
Shared consensus mutations in 16S rRNAs following continuous evolution. An overview of consensus rRNA mutations observed in oRibo-PACE for each starting rRNA species and selection. Values represent % of sequenced clones from each segment (**a**–**c**). **d** Shannon entropy for positions where consensus mutations were discovered in oRibo-PACE. **e** Phylogenetic divergence at positions mutated during oRibo-PACE (outlined squares) show no correlation between a discovered o-rRNA mutation and nucleotide conservation at that position. Shannon entropy values and nucleotide abundance were both obtained from RiboVision^60^. **f** Consensus rRNA mutations discovered in PACE and their locations on the ribosome. Most ribosomal proteins have been omitted for clarity. A close-up view of h37 in the 16S rRNA and the C1098U mutation in relation to ribosomal protein uS2. Close-up locations of U409C (*V. cholerae* only) and C440U (*P. aeruginosa* only) mutations in relation to ribosomal protein uS4. And a close-up view of mutations discovered by ≥2 rRNA evolution campaigns on h27 and h44 in relation to uS12. For all parts, images were generated from a 2.8-Å *Thermus thermophilus* 70S ribosome structure (PDB 4v51^61^). All positions are numbered using *E. coli* 16S rRNA nomenclature. Source data are available in the Source Data File.

Individual clone sequencing at the end of each segment revealed sweeping mutations in all SP-borne o-rRNAs (Supplementary Fig. 3i and Supplementary Tables 1–3). Collectively, *V. cholerae* o-rRNAs developed the highest average number of mutations per clone throughout all segments, while *P. aeruginosa* o-rRNA retained the lowest number of mutations (Fig. 3e). This trend is consistent with propagation efficiencies of the corresponding SPs during oRibo-PACE (Fig. 3d). A number of unique mutations became prevalent in each SP population at varying segments: C1098U (*E. coli*, S3), G1415A (*E. coli*, S1), U409C (*V. cholerae*, S3), and A434U (*P. aeruginosa*, S1) (Fig. 4a–c and Supplementary Fig. 3i, Supplementary Tables 1–3). Interestingly, we note varying levels of natural sequence conservation at the discovered sites (Fig. 4d, e)^27^, suggesting that mutations at these positions may not necessarily indicate functional relevance.

Notably, an identical mutation in h27 was evolved independently in all o-ribosomes at different segments: A906G in *E. coli* and in *V. cholerae* (S1), and A900G in *P. aeruginosa* (S3) (Fig. 4a–c, f and Supplementary Fig. 3i, Supplementary Tables 1–3). Two identical mutations were also found in the *E. coli* (U904C, G1487A) and *V. cholerae* (U904C, G1488A) populations (Fig. 4a–c, f and Supplementary Fig. 3i, Supplementary Tables 1–3). A906 and U904 (helix 27, *E. coli* numbering) together with G1487 (h44) form an interface with protein uS12 (Fig. 4f) during tRNA selection and ensure translation accuracy^28–30^. The observation of three converged mutations (U904C, A906G, and G1487A) in the *E. coli* and *V. cholerae* populations suggests adaptive evolution towards enhanced translational output (Fig. 3d). The *E. coli*-only mutation C1098U (h37) interacts with r-protein uS2^31^ (Fig. 4f) during the final S30 subunit assembly^32^, whereas G1415A is proximal to G1487A (h44, Fig. 4f) and may influence tRNA selection. The *V. cholerae*-only mutation U409C forms a wobble base pair with G433 (h16) interacts with uS4, where its mutation to a cytosine may yield a stronger C409-G433 Watson–Crick pair (Fig. 4f)^31^. The *P. aeruginosa*-only mutation A434U (h17) is near the binding site of protein uS4^3^ (Fig. 4f). Taken together, these results showcase hallmarks of both similar and independent evolutionary trajectories to overcome identical selection regimes.

### PACE-derived o-rRNAs show augmented translation efficiencies

**Fig. 5:**
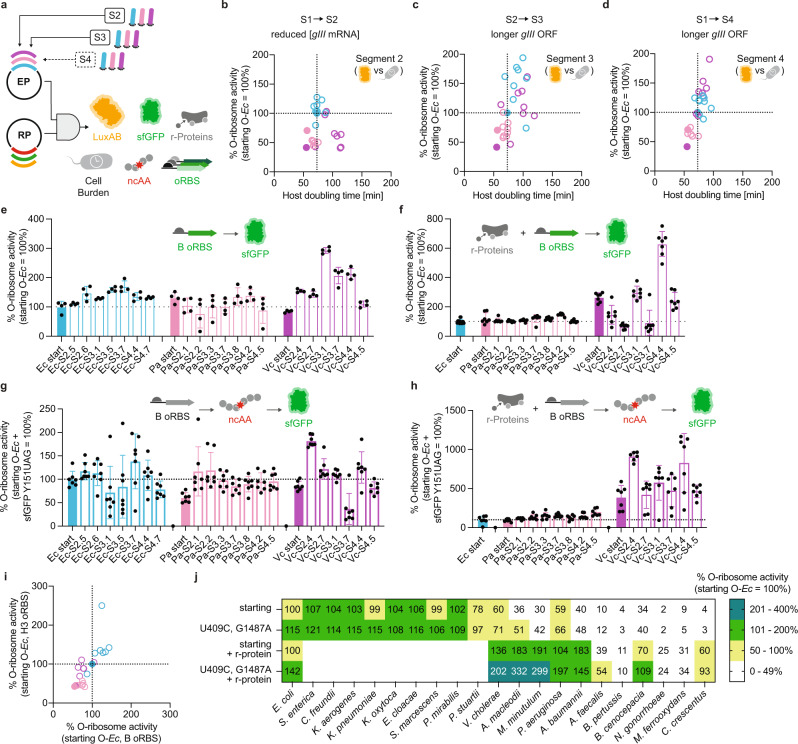
In-depth characterization of evolved o-ribosome activities. **a** o-rRNA variants from each oRibo-PACE segment were cloned into expression plasmids (EPs) and tested alongside reporter plasmids (RPs) of variable genes, RBSs, and context dependencies. Luminescence activity calculated at OD_600_ = 0.15 plotted against host strain (S3489) doubling time is shown for o-rRNA variants derived from each species corresponding to selections; **b** S1→S2, **c** S2→S3, and **d** S1→S4. Data reflect a mean of 1–72 biological replicates (*n* = 1–72). Select o-rRNA variants were prioritized based on luminescence activity and evaluated for sfGFP production in the absence (**e**) or presence (**f**) of cognate heterologous ribosomal proteins (r-proteins). Incorporation of the ncAA BocK for select variants in the absence (**g**) or presence (**h**) of cognate heterologous r-proteins. Data reflect mean and standard deviation of 4–7 biological replicates (*n* = 4–7). **i** sfGFP yield through orthogonal translation and using either the *B* or *H3* o-RBS show comparable activities in most cases. **j** Consensus mutations U409C and G1487 discovered through oRibo-PACE were incorporated into rRNAs derived from phylogenetically divergent bacterial species, and evaluated for sfGFP production in the presence or absence of cognate heterologous r-proteins. In all cases, data are normalized to the activity of the starting *E. coli* o-rRNA activity. Starting rRNAs are shown as filled in bars or circles, whereas evolved variants are shown as borders only. Data reflect the mean and standard deviation of 1–72 biological replicates. Where relevant, data are normalized to the activity of the starting *E. coli* o-rRNA activity (dashed line). Starting rRNAs are shown as filled in bars or circles, whereas evolved variants are shown as borders only. Colors blue (*E. coli*), pink (*P. aeruginosa*), and purple (*V. cholerae*) are consistent across plots. Source Data are available in the Source Data File.

To assess the consequences of PACE-derived mutations on o-ribosome function, we subcloned evolved o-rRNAs into inducible expression plasmids (EPs) and evaluated their activities in vivo using a battery of assays: (1) characterizing translation rate using orthogonal cellular reporter proteins^20^, (2) quantifying host *E. coli* growth burden^33^ during o-ribosome overproduction, (3) investigating possible context-dependence effects on the translation by using the unrelated B o-RBS/o-antiRBS system^8^, (4) analyzing preferential use of *E. coli* host factors by evolved heterologous o-ribosomes via complementation with cognate ribosomal proteins^20^, (5) exploring improvements in genetic code expansion through non-canonical amino acid (ncAA) incorporation^34^, and (6) analyzing context independence of evolved consensus mutations in unrelated, divergent heterologous rRNAs comparing everything to starting *E. coli* o-rRNA under the same conditions (Fig. 5a).

Continuous monitoring of luminescence activity was used as a real-time proxy of the translation rate for o-ribosomes. Using kinetic luminescence output at fixed optical densities (OD_600_ = 0.15, Supplementary Fig. 4a), we observed minimal activity improvements of evolved *E. coli* o-rRNA variants from S2 and S4. However, the six variants isolated from the longer S3 trajectory showed higher (146–196%) activity compared to the starting *E. coli* o-rRNA (Fig. 5b–d and Supplementary Fig. 4b). Only a single *P. aeruginosa* o-rRNA variant evolved after 218 h (S3) of PACE yielded similar activity to starting *E. coli* (96%) luminescence output (Fig. 5c and Supplementary Fig. 4c). Remarkably, almost half (11/24) of *V. cholerae* 16S o-rRNA variants produced higher (109–186%) activities relative to *E. coli* (Fig. 5b–d and Supplementary Fig. 4d). These observed differences in evolved o-rRNA populations, which do not correlate with 16S sequence identity to *E. coli* (*P. aeruginosa*: 85%; *V. cholerae*: 90%), suggest that heterologous rRNA choice may affect directed evolution campaign success by as yet unclear determinants.

Orthogonal ribosomes are known to negatively affect host cell fitness, likely due to over-commitment of resources to the production of supplementary ribosomes (Supplementary Fig. 4e)^20^. We observed a previously reported burden for *E. coli* o-rRNA expression on host cells^33^, and in some cases, these effects are moderately amplified in evolved variants (Fig. 5b–d and Supplementary Fig. 4e–h). In general, host doubling time increased for o-rRNA mutants with respect to starting o-rRNAs (61/67 mutants; 91%), and this trend was held for o-rRNA mutants with enhanced o-ribosome activity as compared to starting scaffold (49/53 mutants; 92.5%) (Fig. 5b–d). However, expressing wild-type or evolved *P. aeruginosa* or *V. cholerae* o-rRNAs exerted a lighter metabolic burden on the *E. coli* host than expressing the corresponding *E. coli* o-rRNAs in many cases (Fig. 5b–d and Supplementary Fig. 4f–h).

Representative variants from each rRNA origin and evolution segments were selected for further evaluation based on kinetic luminescence output. Using an orthogonal superfolder GFP (sfGFP) reporter, we observed the highest o-ribosome activity (sfGFP yield) from *V. cholerae* rRNA mutants (Fig. 5e). Further, we hypothesized that *E. coli* r-proteins may show limited ability to catalyze heterologous ribosome assembly with rRNAs sufficiently divergent to that of *E. coli*, limiting overall functionality of the *P. aeruginosa*- and *V. cholerae*-derived o-rRNAs. To explore this, we complemented o-rRNA variants with cognate r-proteins which we have previously shown can improve heterologous activity^20^. r-Protein complementation of *P. aeruginosa* (using bS16, bS20) and *V. cholerae* (using bS1, uS15, bS16, bS20) o-rRNAs showed greatly increased sfGFP production as compared to the starting *E. coli* o-rRNA, corresponding to 122–147% and 146–629%, respectively (Fig. 5f). These findings show that oRibo-PACE-derived o-rRNAs evolved to overcome the designed selection pressure and did not appreciably adapt to the *E. coli* host.

Orthogonal translation systems have been employed to improve genetic code expansion efforts^1^, yet no reports have extended these capabilities to heterologous ribosomes. We, therefore, evaluated select evolved o-rRNAs for ncAA incorporation by integrating an amber (UAG) stop codon in sfGFP (residue Y151^35^) and assessed Nε-((tertbutoxy)carbonyl)-l-lysine (BocK) incorporation using an established *Methansarcina barkeri*-derived tRNA-synthetase pair^34^. *E. coli*-derived o-rRNA mutants showed no significant increase in BocK incorporation over starting *E. coli* o-rRNA (Fig. 5g). In the absence of cognate phylogenetically divergent r-proteins, *P. aeruginosa* and *V. cholerae* o-rRNA also resulted in negligible improvements in ncAA incorporation over starting *E. coli* (Fig. 5g). However, upon supplementation with cognate r-proteins, *P. aeruginosa* and *V. cholerae*-derived evolved o-rRNAs improved ncAA incorporation efficiency up to 195% and 908%, respectively (Fig. 5h). Context dependence of translation initiation was also evaluated by expressing sfGFP containing either B or H3 o-RBS, where we observed a nearly uniform correlation and clustering by species (Fig. 5i). Interestingly, only *E. coli*-derived o-rRNA variants showed improvements in both B and H3 o-RBS contexts, suggesting that they may have been biased by their initial discovery using *E. coli* o-rRNAs (Fig. 5i).

Finally, we explored the functional relevance of mutations observed with high frequency during the various oRibo-PACE campaigns. Through singular and combinatorial mutations using two unrelated heterologous o-rRNAs (*Salmonella enterica* and *Serratia marcescens* o-rRNAs), we uncovered the combined consensus mutations U409C + G1487A as improving the kinetic capabilities of orthogonal ribosomes (Supplementary Fig. 4i, j). Interestingly, this mutational combination was only observed in the *V. cholerae* campaign, which typically showed greater activities than the *E. coli* and *P. aeruginosa* counterparts across all assays. Both consensus mutations were transplanted into o-rRNAs from increasingly divergent microbes, which resulted in general improvements to translation activities (Fig. 5j). This effect was amplified when tested alongside the cognate r-proteins (Fig. 5j). Excitingly, o-rRNAs from *Alteromonas macleodii* and *Marinospirillum minutulum* increased activity up to 332% and 299 as compared to the starting *E. coli* o-rRNA scaffold, respectively (Fig. 5j). Interestingly, a comparison of wild-type and orthogonal ribosome activities showed that starting *E. coli* o-ribosomes (B o-antiRBS) alongside the cognate reporter gene (B o-RBS) affords a similar protein yield to wild-type *E. coli* ribosomes alongside a wild-type reporter gene in an *E. coli* host (Supplementary Fig. 4k). Accordingly, we normalized all reporter assays to the starting *E. coli* o-rRNA of a given o-RBS context, as enhancements seen within these assays are expected to show improvements over wild-type *E. coli* rRNA. Cumulatively, these extensive analyses demonstrated that oRibo-PACE-derived o-rRNAs enabled the discovery of context-independent mutations that broadly improved o-ribosome activities.

### Kinetically enhanced rRNAs do not enhance population growth

**Fig. 6:**
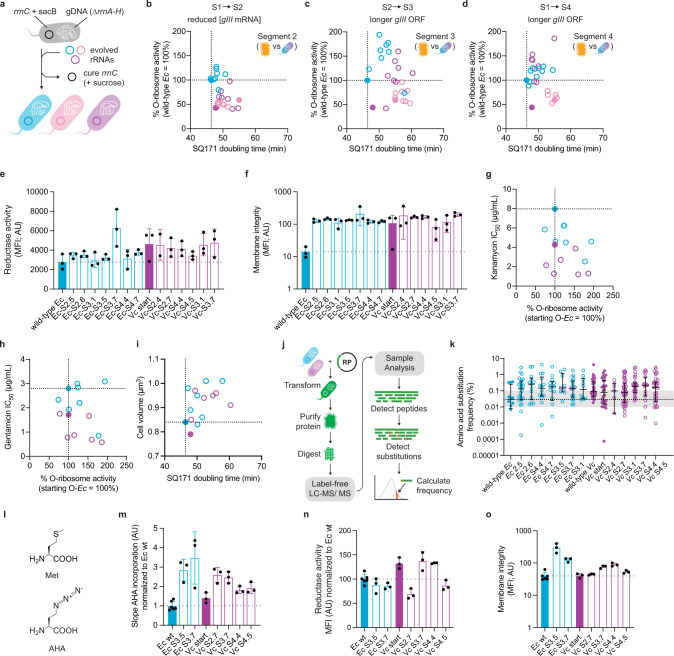
Evolved rRNAs support proteome-wide translation at elevated levels. **a** The o-RBS of oRibo-PACE-derived rRNA variants was substituted with the wild-type RBS, and used to complement SQ171 strains (resident plasmids cured by sucrose selection). O-ribosome luminescence activity plotted against complemented SQ171 strain doubling times for all species corresponding to selections segment: **b** S1→S2, **c** S2→S3, and **d** S1→S4. Data reflect a mean of 1–72 biological replicates (*n* = 1–72). Select rRNA variants were prioritized based on luminescence activity and evaluated for cellular characteristics: electron transport chain function as assessed through cellular reductase activity (**e**) and membrane integrity as assessed through propidium iodide entry (**f**). Data represent mean fluorescence intensity (MFI) with the error shown as the standard deviation of three biological replicates (*n* = 3). SQ171 strain sensitivity to the mistranslation-promoting aminoglycosides kanamycin (**g**) and gentamicin (**h**) negatively correlates evolved o-ribosome activity. **i** Complemented SQ171 strains show increased volume concomitant with observed increases of the population doubling time. **j** Schematic representation of the workflow used to analyze amino acid mistranslation rates through sfGFP purification and LC–MS/MS analysis. **k** The amino acid substitution frequency of select rRNA variants via sfGFP expression, shown as a % of total amino acid detected at a given position. Data reflect sfGFP purified from six pooled biological replicates (*n* = 6). Each point represents an identified amino acid substitution, the horizontal bar represents the median substitution frequency of all misincorporations detected in the sample, and the distribution shown as the interquartile range. The gray bar represents average cellular amino acid mis-incorporation limits. **l** The methionine (Met) analog l-azidohomoalanine (AHA) was used to determine proteome-wide translation rate through unbiased cellular incorporation. **m** Mean slope of AHA incorporation calculated from 20-min time-course analysis. Data normalized to mean slope of wild-type *E. coli* from each experimental run. **n** Complemented SQ171 cells show similar reductase activity and **o** membrane integrity during the AHA incorporation assay. Data reflect the mean and standard deviation of three biological replicates run on different days (*n* = 3). Where relevant, data are normalized to the activity of the starting *E. coli* o-rRNA activity, this is represented as a dashed line. Starting rRNAs are shown as filled in bars or circles, whereas evolved variants are shown as borders only. Colors blue (*E. coli*), pink (*P. aeruginosa*), and purple (*V. cholerae*) are consistent across plots. Source Data are available in the Source Data File.

Analyses of evolved o-rRNA activities suggest that oRibo-PACE can robustly influence ribosome translational kinetics in engineered settings. To elucidate the physiological cost of kinetically enhanced rRNA variants, we introduced the wild-type antiRBS sequence into evolved o-rRNAs and assayed their ability to complement the rRNA efficiency of SQ171 *E. coli* cells and translate all cellular proteins (Fig. 6a and Supplementary Fig. 5a)^25^.

In all cases, evolved 16S rRNAs robustly complemented the ribosomal deficiency of this strain (used alongside native *E. coli* 23S, 5S; Fig. 6b–d and Supplementary Fig. 5b–f). We noted, however, that all evolved variants from oRibo-PACE S3 and S4 that exhibited improved luminescence output (>145% of respective wild-type) showed a concomitant proliferation rate reduction in SQ171 cells (Fig. 6c, d): *E. coli* (6–12% reduction), *P. aeruginosa* (11% reduction), *V. cholerae* (7–24% reduction). *E. coli* ribosome content and therefore translation rate is thought to correlate with cell proliferation^36^, yet kinetically evolved rRNA variants did not result in faster proliferating strains. To further explore this point, all *E. coli* and *V. cholerae* SQ171 strains were assessed for cell vitality in nutrient-rich growth conditions (Davis Rich Medium, DRM)^15^. Analysis of cellular respiration through measurement of electron transport chain function (reductase activity) is a reliable marker of vitality^37^. By assessing the reductase activity and co-staining with propidium iodide, a membrane integrity marker, using all *E. coli* and *V. cholerae* mutants, we observed comparable reductase activity between all strains (Fig. 6e) with indications of minor compromises in membrane integrity as compared to wild-type *E. coli* (Fig. 6f). We did not pursue further analyses using *P. aeruginosa* derived ribosomes due to poor overall activities across most assays.

We hypothesized that the observed reduction in membrane integrity and cell population growth may derive from protein mistranslation by evolved rRNAs. Whereas perturbation of translation rates through ribosomal protein (rpsD, rpsE) mutations can impact the fidelity of protein synthesis^5^, to our knowledge no such relationship between speed and fidelity has been identified for kinetically enhanced translation. To explore this relationship, we first tested complemented SQ171 strains for sensitivity to aminoglycosides as a marker of amino acid mis-incorporation^38^. Interestingly, we find that sensitivity to the aminoglycosides kanamycin and gentamicin correlated negatively with *E. coli*- (Pearson correlation coefficient, or PCC = −0.6086) and *V. cholerae*-derived variants (PCC = −0.5248) (Fig. 6g, h and Supplementary Fig. 6a, b). SQ171 strains encoding evolved rRNAs also showed an increase in overall cell volume that positively correlated with a population doubling time (Fig. 6i), suggesting that kinetically enhanced ribosomes may impact cell size by accumulating proteins at a non-physiological rate, thereby impacting the balance between cell growth and division^39^. We note, however, that increased cell volume under nutrient-rich growth conditions is correlated with a higher average cell growth rate^40^, yet this relationship was absent for our kinetically evolved rRNA variants.

Motivated by these observations, we investigated the translational fidelity of evolved rRNAs. Complemented SQ171 strain-derived sfGFP was subjected to trypsinization and label-free LC–MS/MS to quantify amino acid mis-incorporation (substitution) events (Fig. 6j)^41^. For strains encoding wild-type *E. coli* and the starting *V. cholerae* strain rRNAs, we observed a median amino acid substitution frequency between 1 × 10^−3^ and 10^−4^, suggesting these ribosomes translate with natural tolerable error rates (Fig. 6k). All *E. coli*-derived and 4/6 tested *V. cholerae*-derived mutants, display median amino acid substitution frequencies above ≥2 × 10^−3^ (Fig. 6k), although we do not observe a strong correlation between o-rRNA activity (Supplementary Fig. 6c). Interestingly, specific regions of the sfGFP transcript were enriched in mistranslation events in an o-rRNA-independent manner (Supplementary Fig. 6d, e), although no clear codon ambiguity or amino acid mistranslation preference emerged from these analyses (Supplementary Fig. 6f).

PACE-evolved o-rRNA variants typically showed an enhanced translation rate over starting *E. coli* rRNA under various reporter gene and o-RBS contexts (Fig. 5). To investigate whether these observations extended to proteome-wide translation, we quantified the relative translation rates of complemented SQ171 strains through l-azidohomoalanine (AHA) (Fig. 6l) incorporation in defined minimal medium and quantification following click-chemistry labeling^42^. We observed higher average translation rates in *V. cholerae* mutants (S2.7, S3.7) and *E. coli* mutants (S3.5, S3.7) as compared to *E. coli* and *V. cholerae* starting ribosomes (Fig. 6m). Analysis of viable cells revealed an average AHA incorporation rate increase of >2-fold by *Vc* mutants S2.7 and S3.7 as compared to wild-type *E. coli* (Fig. 6m). Under these conditions, we observed comparable degrees of reductase activity between all strains (Fig. 6n) and slightly decreased membrane integrity in the *Vc* mutant S3.7 and S4.4 strains (Fig. 6o). Overall, these data showcase the ability of the discovered mutations to impart enhanced kinetic properties to ribosomes in native settings, and that faster translation results in a moderate reduction in translational fidelity. These data indicate that ribosome kinetic potential is not maximized but rather refined within a cellular context to balance translation rate and error.

## Discussion

Established models intimately link bacterial ribosome content, proteome-wide protein synthesis rate, and population proliferation rate^43^. Given its critical role in modulating cellular growth rate, the ribosome content of a cell is tightly regulated to mitigate over-commitment of resources^36^. Indeed, rRNAs and ribosomal proteins (r-proteins) can make up approximately half of the total *E. coli* dry mass^44^. Bacteria encoding more ribosomal RNA (rRNA) operons often show increased growth rates^45^ and r-proteins are synthesized preferentially over all other proteins during exponential growth^46^, suggesting that increases in the cellular commitment to ribosome production may dictate the growth rate of a cell. In addition to ribosome content, cellular translation rate may be influenced by a multitude of other factors, including the translation initiation efficiency^47^, aminoacylated tRNA abundance^48^, elongation factor availability^49^, messenger RNA (mRNA) codon usage^50^, and amino acid composition of the nascent polypeptide^51^. Whereas reduction of ribosome elongation rate can negatively impact bacterial proliferation rates^52^, it has remained unclear if kinetically enhanced ribosomes would result in a correspondingly amplified protein yield and rapid population growth. Curiously, reduced translation kinetics can enhance the fidelity of protein synthesis^51^, suggesting some interplay between these two parameters. Here, we describe the first efforts, to our knowledge, to enhance ribosome translation rates above natural speeds.

To explore the kinetic potential of translation, we envisioned that ribosome-directed evolution would provide access to genotypes with faster-than-natural translation rates. To overcome inherent challenges in ribosome-directed evolution, we developed oRibo-PACE, which combines in vivo orthogonal translation^6^ and phage-assisted continuous evolution^10^. To afford this system, we systematically optimized parameters of the platform known to affect the efficiency of orthogonal translation: (1) o-RBS/o-antiRBS interactions to limit crosstalk with host ribosomes, (2) sensor plasmid architecture to enhance orthogonal translation sensitivity, and (3) deletion of host hibernation factors that we show, for the first time, can limit orthogonal translation capabilities. These advances yield an orthogonal translation system that supports phage propagation with high efficiency and minimal crosstalk (>70,000-fold above background). We validated this system using orthogonal *Escherichia coli*-derived ribosomes, and extended these capabilities to two related heterologous ribosomes from *P. aeruginosa* and *V. cholerae*^20^.

Convergent two and three-stage oRibo-PACE selection regimes yielded o-rRNA variants with putatively enhanced kinetic activity above starting rRNA scaffolds. We validated representative variants using multiple reporter genes, o-RBS/antiRBS pairs, and r-proteins complements, finding context-independent improvements in protein translation. Interestingly, we discovered consensus mutations at positions known to interact with ribosomal proteins uS2, uS4, and uS12, all of which play roles in tRNA selection (Fig. 4)^28,29,53^, and suggesting general solutions for rapid translation using diverse o-rRNA scaffolds. However, we noted some discrepancies between activities of evolved heterologous o-rRNA variants that cannot be exclusively attributed to phylogenetic distance from *E. coli*, but may reflect incompatibility between heterologous components and the host translational machinery. In some cases, PACE-derived variants had contrasting effects in orthogonal translation and SQ171 complementation assays, indicating that requirements for these activities may not be fully congruent.

Nonetheless, we find that oRibo-PACE-evolved ribosomes imparted improved translation activities across a myriad of assays, and in both native and orthogonal translation contexts. In particular, orthogonal translation system development has maintained a long-standing goal of improving the decoding of nonsense codons and or incorporation of ncAAs for genetic code expansion^1^. Combining oRibo-PACE discovered consensus mutations and heterologous ribosome engineering achieved 630% reporter protein yield (Fig. 5f). This same combination of ribosome evolution and engineering improved the incorporation of ncAAs within our reporter protein by 908% over the current state-of-the-art orthogonal translation system (Fig. 5h). We expect that our findings, in particular, that starting rRNA templates can greatly impact selection outcome, will inform approaches for ribosome engineering and directed evolution. In addition, future-directed evolution efforts that integrate selections for translational fidelity may provide greater insight into the kinetics upper limit tolerated by living cells. Using methods and resources described in this study, we envision the development of robust capabilities for improved protein biomanufacturing and genetic code expansion (DeBenedictis et al., submitted), explorations of cell growth regulation, and illumination of the fundamental structure–function relationships within the ribosome.

## Methods

### Bacterial strains

All DNA manipulations were performed using NEB Turbo cells (New England Biolabs) or Mach1F cells, which are Mach1 T1^R^ cells (ThermoFisher Scientific) mated with S2057 F′ to constitutively provide TetR and LacI. All infection assays, plaque assays, and PACE experiments were performed with *E. coli* S3317 or S3489 as indicated. Both strains were derived from *E. coli* S2060^13^ and modified using the recombineering method^54^ as follows: (i) scarless deletion of hibernation promoting factor (HPF)^21^ to reduce rRNA inactivation; (ii) deletion of *fhuA*, a lytic bacteriophage entry receptor^24^, to facilitate turbidostat PACE experiments.

### DNA cloning

Water was purified using a MilliQ water purification system (Millipore). Genes were amplified by PCR from native sources as previously described^20^. All plasmids and selection phages were constructed using USER cloning^55^. Briefly, a single internal deoxyuracil base was included at 15–20 bases from the 5′ end of the primer. This region is described as the USER junction, which specifies the homology required for correct assembly. USER junctions were designed to contain minimal secondary structure, have 42 °C < *T*_m_ < 70 °C, and begin with a deoxyadenosine and end with a deoxythymine (to be replaced by deoxyuridine). Phusion U Hot Start DNA Polymerase (Life Technologies) is used in primers carrying deoxyuracil bases. MinElute PCR Purification Kit (Qiagen) was used to purify all PCR products to 10 μl final volume, which was quantified using a NanoDrop 1000 Spectrophotometer (ThermoFisher Scientific). For USER assembly, an equimolar ratio (up to 1 pmol each) of PCR products carrying complementary USER junctions were mixed in a 10 μl reaction containing 0.75 units DpnI (New England Biolabs), 0.75 units USER (Uracil-Specific Excision Reagent; Endonuclease VIII and Uracil-DNA Glycosylase) enzyme (New England Biolabs), 1 unit of CutSmart Buffer (50 mM potassium acetate, 20 mM Tris-acetate, 10 mM magnesium acetate, 100 μg ml^−1^ BSA at pH 7.9; New England Biolabs). The reactions were incubated at 37 °C for 20 min, followed by heating to 80 °C and slow cooling to 4 °C at 0.1 °C s^−1^ in a thermocycler. The hybridized constructs were directly used for heat-shock transformation of chemically competent NEB Turbo *E. coli* cells or Mach1F *E. coli* cells. Agar-2xYT plates (1.8%; United States Biological) supplemented with the appropriate antibiotic(s) were used to select for transformants.

For selection phage cloning, the hybridized constructs were transformed into chemically competent S3489 cells carrying the accessory plasmid pJC175e^15^, where pIII is produced in response to phage infection. After recovery for 12 h at 37 °C at 300 rpm in 2xYT media (United States Biological), the culture was centrifuged for 2 min at 10,000 × *g* and the supernatant was purified using 0.22 μm PVDF Ultrafree centrifugal filter (Millipore). The titer of each clonal phage stock was determined through plaque assays (see the section below). In all cases, cloned plasmids and phages were verified by Sanger sequencing using a template generated using the TempliPhi 500 Amplification Kit (GE Life Sciences) according to the manufacturer’s protocol.

### Plaque assays

S3317 or S3489 cells carrying the accessory plasmid of interest were grown at 37 °C to OD_600_ = 0.6–0.9 in 2xYT (United States Biological) liquid media supplemented with the appropriate antibiotics. The stock of phage supernatant was filtered using a 0.22 μm PVDF Ultrafree centrifugal filter (Millipore Sigma) and diluted in three, 100-fold serial dilution increments to yield four total samples (undiluted, 10^2^-, 10^4^-, and 10^6^-fold diluted). For each sample, 10 μl of phage was added to a sample library tube (VWR). S3317 or S3489 cells carrying the accessory plasmid of interest were grown at 37 °C to OD_600_ = 0.6–0.9 in 2xYT (United States Biological) liquid media supplemented with the appropriate antibiotics. Next, 150 μl of cells were added to each library tube containing phage. Within 1–2 min of infection, 1 ml of warm (~55 °C) top agar (0.4% agar-2xYT) supplemented with 0.04% Bluo-Gal (Gold Biotechnology) was added to the phage/cell mixture. After mixing by pipetting up and down once, each 1.16-ml mixture was plated onto one quadrant of a quartered plate with 2 ml of bottom agar (1.8% agar-2xYT). After solidification of the top agar, the plates were grown overnight (~18 h) at 37 °C before plaques, stained blue following Bluo-Gal cleavage, could be observed.

### Enrichment assays

S3317 or S3489 cells carrying the accessory plasmid of interest were grown in Davis rich media (DRM) media^15^ supplemented with the appropriate antibiotics to OD_600_ = 0.2. The SP supernatant was added to a final titer of 10^5^ pfu ml^−1^ and grown for 14–18 h in a 37 °C shaker at 300 rpm. Cultures were centrifuged using a table-top centrifuge for 2 min (10,000 × *g*). The supernatant was filtered through a 0.22 μm PVDF Ultrafree centrifugal filter (Millipore Sigma) and titered by plaque assay on S3317 or S3489 cells with pJC175e (total phage titer), S3317 or S3489 cells with ^proC^AP3_H3-1_ (activity-dependent phage titer, pAB171c), and/or S3317 or S3489 cells without any AP (recombinant M13-like SP titer). If necessary, purified phage samples were stored overnight at 4 °C prior to plaquing.

### PACE

Host cell cultures, lagoons, media, and the PACE apparatus were prepared as previously described^10^. Briefly, MP6^18^ was co-transformed into chemically competent S3489 or S3317 cells alongside the AP of interest and recovered for 45 min at 37 °C using DRM supplemented with 25 mM d-fucose to ensure MP repression via catabolite repression by glucose (a component of DRM) and competitive inhibition of araC by d-fucose^56,57^. Transformations were selected on 1.8% agar-2xYT plates containing kanamycin (30 μg ml^−1^), chloramphenicol (40 μg ml^−1^), 25 mM glucose (United States Biological), and 25 mM d-fucose (Carbosynth). After incubation at 37 °C for 12–18 h, six individual colonies were picked, resuspended in DRM, 10-fold serially diluted and plated on 1.8% agar-2xYT plates with kanamycin (30 µg ml^−1^), chloramphenicol (40 μg ml^−1^) and containing either 25 mM arabinose (Gold Biotechnology) or 25 mM glucose and 25 mM d-fucose. After incubation for 12–18 h at 37 °C, the plates were examined to confirm arabinose sensitivity. Concomitant with the aforementioned plating step, the resuspended colonies and dilutions thereof were used to inoculate liquid cultures in DRM supplemented with kanamycin (30 μg ml^−1^), chloramphenicol (40 μg ml^−1^), 25 mM glucose, and 25 mM d-fucose. The cultures were grown in a 37 °C shaker at 900 rpm (Infors HT Multitron Pro) to OD_600_ = 0.2, at which point 1-ml of cells were added directly to 300 ml of fresh DRM in the turbidostat. The turbidostat culture was maintained at 300 ml and optical density was maintained at OD_600_ = 0.8–0.9 using a TruCell2 probe^10^. All lagoons supplied by the turbidostat were maintained at 40 ml, and diluted as described previously^10^. Prior to infection with SPs, lagoons were supplemented to a final concentration of 25 mM arabinose using a syringe pump (New Era Pump Systems) for 1 h to induce the MP. At the indicated time points, samples were collected from each lagoon and SP was purified as described above.

### Mutagenesis during PACE

The basal mutation rate of replicating filamentous phage in *E. coli* is 7.2 × 10^−7^ substitutions per base pair per generation, which is sufficient to generate all possible single but not double mutants of a given 1000 base pair gene in a 40-ml lagoon after one generation of phage replication. For the 16S ribosomal subunit (1542 base pairs), a basal mutation rate of 7.2 × 10^−7^ substitutions per base pair per generation applied to 2 × 10^10^ copies of the gene (a single generation in a 40-ml lagoon) yields ~2.2 × 10^7^ base substitutions (7.2 × 10^−7^ substitutions per base pair * 1542 base pairs * 2 × 10^10^ copies), which could cover all 4.6 × 10^3^ single point mutants and all ~2.1 × 10^7^ double point mutants. Arabinose induction of the high-potency mutagenesis plasmid MP6^18^ increases the phage mutation rate to 7.2 × 10^−3^ substitutions per base pair per generation, yielding ~2.2 × 10^11^ substitutions spread over 2 × 10^10^ copies of the gene after a single generation. This elevated mutation rate is sufficient to cover all possible single mutants (4.6 × 10^3^ possibilities), double mutants (2.1 × 10^7^ possibilities), and triple mutants (9.9 × 10^10^ possibilities) after a single phage generation.

### Luminescence assays

Log-phase (OD_600_ = 0.3–0.5) S3489 cells carrying the reporter plasmid (RP) pFL19c grown in 2xYT (United States Biological) was made chemically competent, later transformed with the desired EP, and recovered for 2 h in Terrific Broth (Millipore Sigma). All transformations were plated on 1.8% agar-2xYT plates (United States Biological) supplemented with kanamycin (30 µg ml^−1^) and carbenicillin (50 µg ml^−1^). The plates were incubated for 12–18 h in a 37 °C incubator. Colonies transformed with the appropriate EP were picked the following day and grown in DRM-containing kanamycin (30 µg ml^−1^) and carbenicillin (50 µg ml^−1^) for 18 h. Following overnight growth of the EP/RP-carrying strains, cultures were diluted 100-fold into fresh DRM supplemented with kanamycin (30 µg ml^−1^) and carbenicillin (50 µg ml^−1^). The cultures were induced with anhydrotetracycline (1000 ng ml^−1^), and 200 μL of each culture was transferred to a 96-well black wall, clear bottom plate (Costar), and topped with 20 µl of mineral oil (Millipore Sigma). OD_600_ and luminescence values for each well were monitored using an Infinite M1000 Pro microplate reader (Tecan) over 15 h. Each variant was assayed in 8–24 biological replicates. Luminescence activities were tabulated at OD_600_ = 0.15 in all cases.

### Fluorescent protein assays

Chemically competent 3489 were transformed with ribosome expression plasmid (EP) and desired reporter plasmid (RP), and recovered for 2 h in Terrific Broth (Millipore Sigma). All transformations were plated on 1.8% agar-2xYT plates (United States Biological) supplemented with kanamycin (30 µg ml^−1^) and carbenicillin (50 µg ml^−1^). The plates were incubated for 12–18 h in a 37 °C incubator. Colonies transformed with the appropriate EP were picked the following day and grown in DRM-containing kanamycin (30 µg ml^−1^), carbenicillin (50 µg ml^−1^), and anhydrotetracycline (1000 ng ml^−1^). After growth for 16–24 h at 37 °C with 900 rpm shaking, 200 μl of each culture was transferred to a 96-well black wall, clear bottom plate (Costar), and topped with 20 µl of mineral oil (Millipore Sigma). OD_600_ and fluorescence values (excitation at 485 nm, emission at 510 nm) for each well were monitored using an Infinite M1000 Pro microplate reader (Tecan) or Spark plate reader (Tecan). Each variant was assayed in 4–8 biological replicates. Fluorescent protein yields were normalized to culture OD_600_ in all cases.

### ncAA incorporation assays

Chemically competent 3489 were transformed with complementary plasmid (CP) pTECH Mb PylRS IPYE^34^ with resistance changed for DHFR, and desired EP and RP (pAB140g (WT sfGFP) or pAMC025a (UAG151 sfGFP) or pAMC016a (WT luxAB) or pAMC016b (UAG luxAB)), and recovered for 2 h in Terrific Broth (Millipore Sigma). All transformations were plated on 1.8% agar-2xYT plates (United States Biological) supplemented with trimethoprim (3 µg ml^−1^), kanamycin (10 µg ml^−1^), and carbenicillin (15 µg ml^−1^). The plates were incubated for 12–18 h in a 37 °C incubator. Colonies transformed with the appropriate EP and RP were picked the following day and grown in DRM-containing trimethoprim (3 µg ml^−1^), kanamycin (10 µg ml^−1^), and carbenicillin (15 µg ml^−1^) for 20–24 h. Overnight cultures were 100-fold for luminescence assays in fresh DRM containing; (3 µg ml^−1^), kanamycin (10 µg ml^−1^), carbenicillin (15 µg ml^−1^), anhydrotetracycline (40 ng ml^−1^), with or without Nε-((tertbutoxy)carbonyl)-l-lysine (BocK) (1 mM) (Bachem). For sfGFP assays, colonies were picked directly into complete assay media. OD_600_ and luminescence values for each assay were monitored using an Infinite M1000 Pro microplate reader (Tecan) or Spark plate reader (Tecan). Each variant was assayed in 4–8 biological replicates. Luminescence activities were tabulated at OD_600_ = 0.15 in all cases. Fluorescent protein yield was normalized to culture OD_600_ at saturation (OD_600_ ~ 1.5).

### SQ171 complementation assays

Log-phase (OD_600_ = 0.3–0.5) cells of SQ171^25,58^ grown in 2xYT (United States Biological) were transformed with the desired EP, and recovered for 5 h in 2xYT in a 37 °C shaker. The recovery culture was centrifuged at 10,000 × *g* for 2 min, then the pellet was resuspended in 100 µl 2xYT. The resuspended cells were diluted serially in seven, 10-fold increments to yield eight total samples (undiluted, 10^1^-, 10^2^-, 10^3^-, 10^4^-, 10^5^-, 10^6^-, and 10^7^-fold diluted). To determine the efficiencies of EP transformation and counter-selectable plasmid curing, 3 μl of each sample of the diluted series were plated on 1.8% agar-2xYT plates (United States Biological) supplemented with spectinomycin (100 μg ml^−1^) and carbenicillin (50 μg ml^−1^), with or without 5% sucrose (Millipore Sigma). For picking single colonies, the remaining undiluted cells were plated on 1.8% agar-2xYT plates (United States Biological) containing spectinomycin (100 μg ml^−1^), carbenicillin (50 μg ml^−1^), and 5% sucrose. All plates were grown for 12–18 h in a 37 °C incubator. Colonies transformed with the appropriate EP and surviving sucrose selection were picked and grown in DRM-containing spectinomycin (100 μg ml^−1^), carbenicillin (50 μg ml^−1^), and 5% sucrose. Following overnight growth of the EP-carrying strains, cultures were diluted 250-fold into fresh DRM-containing spectinomycin (100 μg ml^−1^) and carbenicillin (50 μg ml^−1^). From the diluted cultures, 150 μl of each culture was transferred to a 96-well black wall, clear bottom plate (Costar), topped with 20 µl of mineral oil, and the OD_600_ was measured every 5 min over 15 h. Separately, 400 µl of each diluted culture was supplemented with kanamycin (30 µg ml^−1^) and grown in a 37 °C shaker at 300 rpm. Colonies that survived selection in kanamycin were excluded from the final analysis, as survival in kanamycin indicates the persistence of the resident pCSacB plasmid (which carries a KanR resistance cassette). The doubling time of each culture was calculated using the Growthcurver package (version 0.3.0)^59^ in R (version 3.5.2).

### Cell volume measurement

Complemented SQ171 strains were grown overnight 16–18 h in a 37 °C incubator DRM-containing spectinomycin (100 μg ml^−1^) and carbenicillin (50 μg ml^−1^). Overnight cultures were then diluted 100-fold in fresh DRM-containing spectinomycin (100 μg ml^−1^) and carbenicillin (50 μg ml^−1^). Upon reaching the early log-phase (OD_600_ = 0.1–0.15), cells were diluted 10-fold to synchronize cultures and harvested when the early log-phase was reached again (OD_600_ = 0.1–0.15). Cells were then placed on ice and cell volumes were measured in filtered PBS (0.2 µm filter) using Coulter Counter (Beckman Coulter) with a 20 µm aperture. Particles smaller than 0.4 µm^3^ in volume were excluded from the analysis. Measurements were calibrated using NIST traceable 3.0 µm diameter polystyrene beads (ThermoFisher).

### Cell viability and AHA incorporation assays

SQ171 strains were grown for 24 h in M9 minimal media supplemented with all amino acids (defined as M9AA): M9 salts (Teknova), [0.4% w/v] d-glucose, [3.4 mg ml^−1^] thiamine hydrochloride, [1 mM] MgSO_4_, [0.25 mM] CaCl_2_, [1.33 mg l^−1^] amino acid mix (-Methionine) (MANU), 200 µM l-Methionine (Sigma Aldrich), spectinomycin (100 μg ml^−1^) and carbenicillin (50 μg ml^−1^). Overnight cultures were diluted 100-fold in fresh M9AA and grown to OD_600_ = 0.1–0.15. Cultures were synchronized by diluting once more by 10-fold and continuing to grow. At OD_600_ = 0.1–0.15, cultures were harvested by centrifugation at 3000 × *g* for 5 min, media was exchanged for M9AA-M (defined as M9AA excluding l-methionine), and cultures returned to 37 °C incubator at 300 rpm. After 1 h, M9AA-M outgrowth cells were treated with 200 µM l-azidohomoalanine (AHA) (Click Chemistry Tools) and BacLight RedoxSensor Green Vitality Kit reagents (Invitrogen) per the manufacturer protocols for 5, 10, 15, 20, or 30 min at 37 °C and 300 rpm. AHA incorporation was blocked at each time interval by adding 200 µg ml^−1^ chloramphenicol, whereas RedoxSensor Green was blocked at each time interval by adding 10 mM NaN_3_. Negative control samples for AHA incorporation and cell vitality were treated with 200 µg ml^−1^ chloramphenicol or 10 mM NaN_3_, respectively, 10 min prior to AHA or RedoxGreen addition. Following AHA incorporation and vitality labeling, cells were washed using 0.5 ml PBS, fixed in 3.8% PFA for 10 min at room temperature, washed twice with PBS, permeabilized with 0.2% Triton X-100 for 10 min in RT, and washed twice more in PBS. Samples were stored at 4 °C for subsequent Click-IT chemistry. Fixed and permeabilized cell samples were mixed with Click-&-Go Cell Reaction Buffer (Click Chemistry Tools) containing 2.5 µM AlexaFluor 405 Alkyne (Click Chemistry Tools) according to manufacturer instructions, and were incubated for 30 min in the dark at room temperature, then washed twice with PBS. Labeled cells were analyzed with BD Biosciences flow cytometer LSR II HTS with excitation lasers at 405, 488, and 561 nm and emission filters at 450/50, 515/20, and 610/20 nm. Cells were gated on forward and side scatter, and particles/cells with minimal vitality labeling were excluded. The AHA incorporation rate represents the rate of linear increase in population mean AHA incorporation over 20 min. For viability assays not investigating AHA incorporation, strains were grown in DRM. Overnight cultures were diluted 100-fold in fresh M9AA and grown to OD_600_ = 0.1–0.15. Cultures were again synchronized by diluting once more by 10-fold and continuing to grow. At OD_600_ = 0.1–0.15, cultures were labeled with BacLight RedoxSensor Green Vitality Kit reagents (Invitrogen) per the manufacturer protocols, for 30 min at 37 °C with 300 rpm shaking. Flow data were analyzed using FlowJo v10.

### Aminoglycoside sensitivity assays

SQ171 strains carrying wild-type or evolved rRNA variants were grown in DRM-containing spectinomycin (100 μg ml^−1^) and carbenicillin (50 μg ml^−1^) for 12–18 h. Overnight cultures were diluted 50-fold in fresh DRM-containing spectinomycin (100 μg ml^−1^), carbenicillin (50 μg ml^−1^), and mixed 1:1 with a dilution series of kanamycin or gentamicin (64, 32, 16, 8, 4, 2, 1, 0.5, 0.25 μg m^l−1^). Cultures were grown at 37 °C with shaking, 900 rpm, overnight for 24 h. OD_600_ for each well was quantified using an Infinite M1000 Pro microplate reader (Tecan). IC_50_ values for kanamycin and gentamicin resistance of each strain were calculated in Prism (v 9.1.0).

### Protein purification

SQ171 strains transformed with pED17x1 (sfGFP with C-terminal His-tag) were lysed by B-per (ThermoFisher), 4 ml per gram weight of the pellet. To each sample, 120 µl of B-per + protease inhibitor (Roche) was added and incubated for 1 h at room temperature with gentle rocking. Soluble protein was fractionated by centrifugation at 16,000 × *g* for 20 min and removing supernatant (soluble protein). 300 µl of each sample was loaded onto a His-Spin Protein Mini-prep column (Zymo) and purified using the manufacturer’s protocol. All samples were eluted in 150 µl of elution buffer. Gel-code blue-stained SDS–PAGE gel lanes were subdivided into 7 regions and cut into ~2 mm squares. These were washed overnight in 50% methanol/water. These were washed once more with 1:1 methanol:water overnight, dehydrated with acetonitrile and dried in a speed-vac. Reduction and alkylation of disulfide bonds were then carried out by the addition of 30 µl 10 mM dithiothreitol (DTT) in 100 mM ammonium bicarbonate for 30 min to reduce disulfide bonds. The resulting free cysteine residues were subjected to an alkylation reaction by removal of the DTT solution and the addition of 100 mM iodoacetamide in 100 mM ammonium bicarbonate for 30 min to form carbamidomethyl cysteine. These were then sequentially washed with aliquots of acetonitrile, 100 mM ammonium bicarbonate, and acetonitrile and dried in a speed-vac. The bands were enzymatically digested by the addition of 300 ng of trypsin (or chymotrypsin for R or K qtRNAs) in 50 mM ammonium bicarbonate to the dried gel pieces for 10 min on ice. Depending on 22the volume of acrylamide, excess ammonium bicarbonate was removed or enough was added to rehydrate the gel pieces. These were allowed to digest overnight at 37 °C with gentle shaking. The resulting peptides were extracted by the addition of 50 µl (or more if needed to produce supernatant) of 50 mM ammonium bicarbonate with gentle shaking for 10 min. The supernatant from this was collected in a 0.5 ml conical autosampler vial. Two subsequent additions of 47.5/47/5/5 acetonitrile/water/formic acid with gentle shaking for 10 min were performed with the supernatant added to the 0.5 ml autosampler vial. The organic solvent was removed and the volumes were reduced to 15 µl using a speed-vac for subsequent analyses.

### Chromatographic separations and analysis

Digested extracts were analyzed by reversed-phase high-performance liquid chromatography (HPLC) using Waters NanoAcquity pumps and autosampler and a ThermoFisher Orbitrap Elite mass spectrometer using a nanoflow configuration. A 20 mm × 180 µm column packed with 5 µm Symmetry C18 material (Waters) using a flow rate of 15 µl min^−1^ for 3 min was used to trap and wash peptides. These were then eluted onto the analytical column which was a self-packed with 3.6 µm Aeris C18 material (Phenomenex) in a fritted 20 cm × 75 µm fused silica tubing pulled to a 5 µm tip. The gradient was isocratic 1% A Buffer for 1 min 250 nl min^−1^ with increasing B buffer concentrations to 15% B at 20.5 min, 27% B at 31 min, and 40% B at 36 min. The column was washed with high percent B and re-equilibrated between analytical runs for a total cycle time of ~53 min. Buffer A consisted of 1% formic acid in water and buffer B consisted of 1% formic acid in acetonitrile.

### Mass spectrometry

The mass spectrometer was operated in a dependent data acquisition mode where the 10 most abundant peptides detected in the Orbitrap Elite (ThermoFisher) using full scan mode with a resolution of 240,000 were subjected to daughter ion fragmentation in the linear ion trap. A running list of parent ions was tabulated to an exclusion list to increase the number of peptides analyzed throughout the chromatographic run. The resulting fragmentation spectra were correlated against custom databases using PEAKS Studio X (Bioinformatics Solutions). Calculation of Limit of Detection and relative abundance. The results were matched to an sfGFP reference and analyzed for ≤2 amino acid substitutions in a single tryptic fragment. The abundance of each residue substitution was quantified by calculating the area under the curve of the ion chromatogram for each peptide precursor. The limit of detection is 10^4^ [AU], the lower limit for the area under the curve for a peptide on this instrument.

### Reporting summary

Further information on research design is available in the Nature Research Reporting Summary linked to this article.

## Supplementary information

Supplementary Information

Reporting Summary

## Data Availability

Key plasmids described in this study will be deposited in Addgene. Unprocessed data files and other plasmids will be made available upon reasonable request. Source data are provided with this paper.

## Source data

Source Data

## Code availability

Code used to analyze codon adaptation index and amino acid mis-incorporation can be accessed via the following link (https://github.com/sbratulic/o-Ribo-PACE).
