# Supplementary material for: Directed evolution of rRNA improves translation kinetics and recombinant protein yield
Source: Nat Commun. 2021 Sep 24;12:5638. doi: 10.1038/s41467-021-25852-5 (PMC8463689; doi:10.1038/s41467-021-25852-5)
Supplement: Supplementary file 1 — Supplementary Information [file 41467_2021_25852_MOESM1_ESM.pdf]

Directed Evolution of rRNA Improves Translation Kinetics and Recombinant Protein Yield

SUPPORTING INFORMATION

Fan Liu[1,*], Siniša Bratulić[1,*], Alan Costello[1,2,*], Teemu Miettinen[3], Ahmed H. Badran[1,2,**]

[1] The Broad Institute of MIT & Harvard University, Cambridge MA 02142, USA.

[2] Department of Chemistry, The Scripps Research Institute, La Jolla CA 92037, USA.

[3] Koch Institute for Integrative Cancer Research, Massachusetts Institute of Technology, Cambridge MA 02142, USA.

* These authors contributed equally: Fan Liu, Siniša Bratulić, Alan Costello

** Correspondence should be addressed to Ahmed H. Badran: ahbadran@broadinstitute.org

ORCID ID:

FL: 0000-0002-4333-7581

SB: 0000-0002-1663-2227

AC: 0000-0002-6266-5070

TM: 0000-0002-5975-200X

AHB: 0000-0002-8105-1883

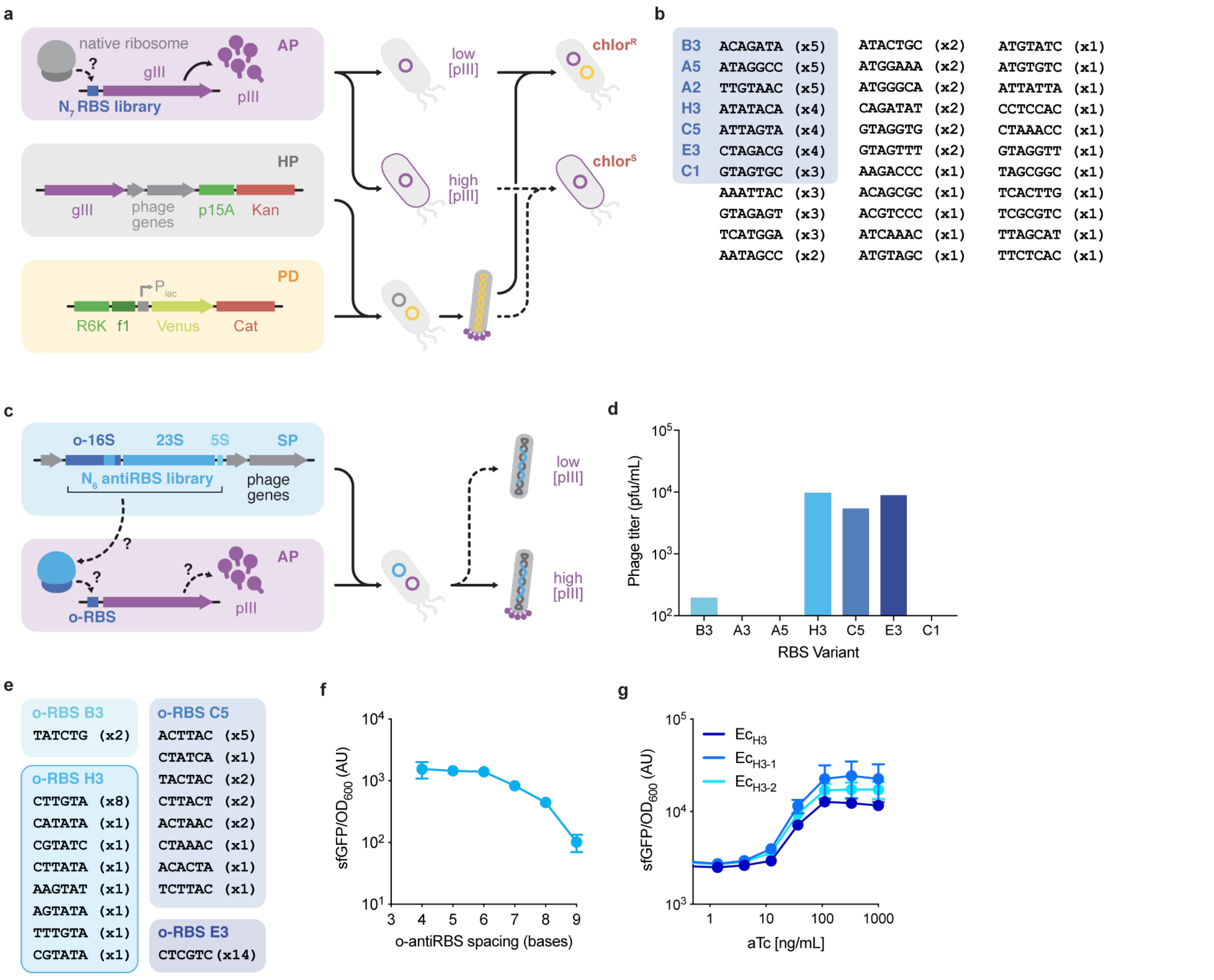

Supplementary Figure 1 | Identification of an optimal o-RBS/o-antiRBS system for SP-borne o-rRNAs. a) A degenerate 4^7 (16,384) member library of RBS variants (o-RBS_{lib}, **Figure 1C**) was introduced to *E. coli* S2060 cells. The resultant cells were infected by phagemids (PDs) carrying the resistance gene for chloramphenicol (*cat*) and packed using a cognate helper phage (HP). APs encoding o-RBSs with significant crosstalk with the host's translational machinery would lead to *gIII* expression, rendering their hosts uninfectable and sensitive to chloramphenicol. O-RBSs with high orthogonality would allow phagemid infection and protection against chloramphenicol. **b)** Sanger sequencing of 96 colonies yielded 33 unique o-RBS sequences, where the number following each sequence indicates frequency of occurrence. The seven most abundant variants (highlighted) were further characterized. **c)** To discover cognate o-antiRBSs, SPs bearing a degenerate library of 4^6 (4,096) antiRBS variants (o-$antiRBS_{lib}$, **Figure 1c)** were used to transform *E. coli* host cells that carry APs encoding each of the seven discovered o-RBSs. Functional o-antiRBS sequences should efficiently translate *gIII*, yielding pIII and giving rise to progeny phage. **d)** The RBS variant H3 (o-RBS_{H3}, **Figure 1c**) provided the most efficient propagation (highest titers) post infection (n = 1). **e)** Sequencing of clonal phage plaques identified up to 8 unique o-antiRBSs for each o-RBS sequence. For o-RBS_{H3}, the CTTGTA sequence (o-$antiRBS_{H3}$, **Figure 1c**) occurred with the highest frequency. **f)** The effect of spacer sequence of o-antiRBS was investigated using an orthogonal sfGFP reporter. The four base pair spacer sequence was found to be optimal and used for all further studies. Data reflects mean and standard deviation of 2-3 biological replicates (n = 2-3). **g)** Experimental validation of two new o-antiRBSs discovered through continuous culturing (**Figure 1c).** Data reflects the mean and standard deviation of 3 biological replicates (n = 3).

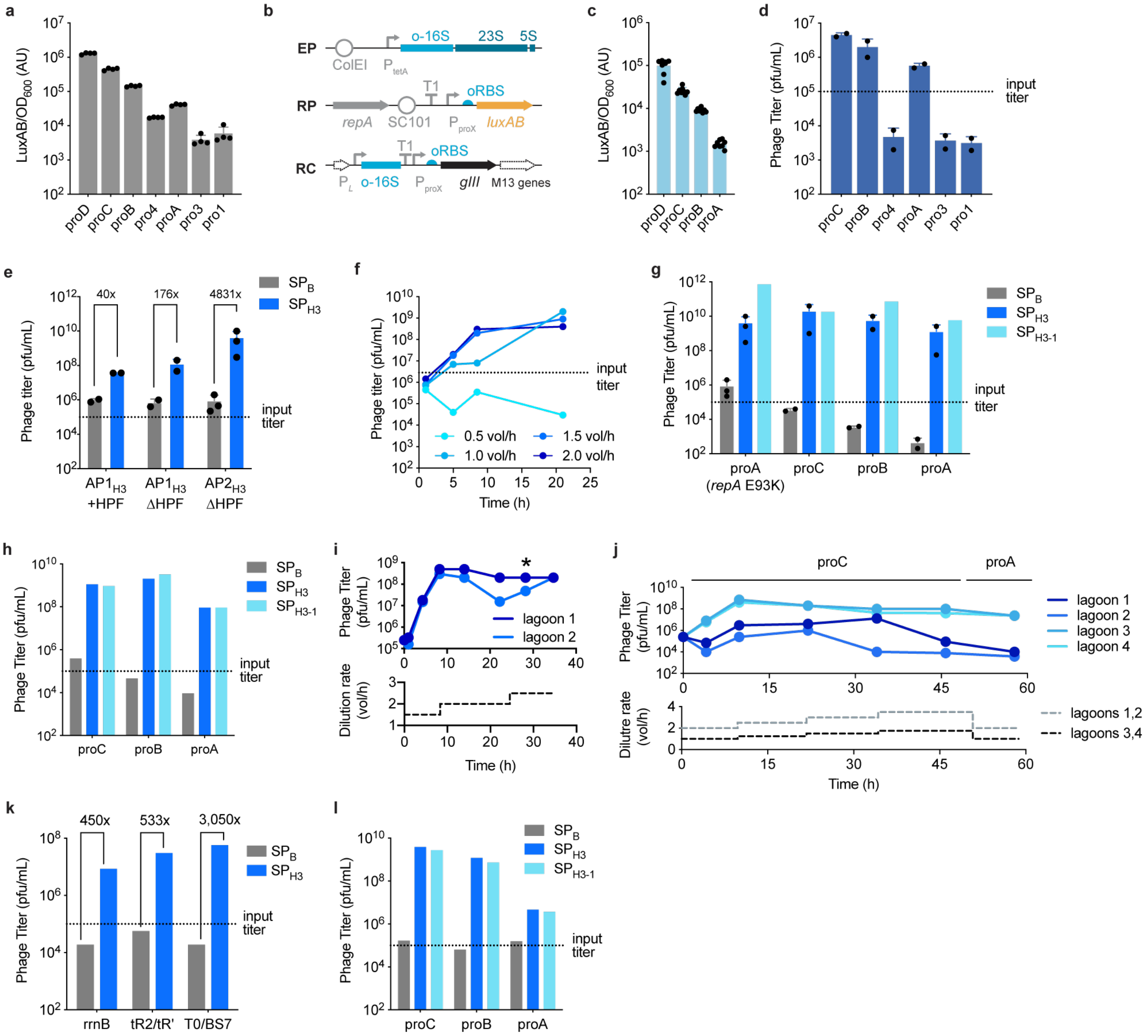

Supplementary Figure 2 | Design and validation of an orthogonal translation-based genetic circuit for continuous directed evolution. Unless otherwise noted, all APs encode the wild-type replication initiation protein RepA (5–7 copies per cell) [1]. RepA bearing the E93K mutation increases plasmid copy number to 26–29 copies per cell [1]. **a)** Comparison of insulated constitutive promoters [2] of varying strengths driving *luxAB* expression. Data reflects mean and standard deviation of 4 biological replicates (n = 4). **b)** Overview of the expression plasmid (EP) and reporter plasmid (RP) used in luminescence assays. A schematic of the sequence-confirmed recombined phage (RC) referenced in **(h)** is also included. **c**) A comparison of luminescence assays using a luciferase reporter with o-RBS_{H3} and an *E. coli* o-ribosome EP. Data reflects mean and standard deviation of 8 biological replicates (n = 8). **d)** Promoter strengths correlate with phage propagation, as demonstrated by a comparison of phage enrichment assays using the constitutive $AP2_{H3}$ (**Figure 1b**) and SP_{H3}. **e)** Comparison of the AP1 and AP2 architectures and the effect of HPF deletion on SP propagation. **f)** O-rRNA-dependent SP propagation is severely attenuated in late stationary phase host cells (under low lagoon flowrates). **g)** A comparison of phage enrichment assays using $AP2_{H3}$ (**Figure 1B**) in S3317 cells using various constitutive promoters. Data reflects mean of 2-3 biological replicates (n = 2-3). **h)** Phage enrichment assays using $AP2_{H3}$ in S3489 cells using various constitutive promoters. S3489 cells are identical to S3317 but have been deleted for the *fhuA* gene to

protect host cells from infections by lytic bacteriophages [3]. **i)** Continuous propagation of SP_{H3-1} using $^{proA}AP2_{H3}$ (RepA E93K; 26–29 copies per cell [2]) in S3317 cells under high mutagenesis conditions using MP6 [4]. M13-like recombinants developed after 28h of continuous culture (indicated by *), which was attributed to high AP copy number. **j)** PACE of SP_{H3-1} using $^{proC}AP2_{H3}$ (wt RepA; 5–7 copies per cell) in S3317 cells showed high phage titers at low lagoon flow rates (t = 0–50 h; lagoons 3 and 4) but SP washout occurred at high lagoon flow rates (t = 0–50 h; lagoons 1 and 2). The combination of wt RepA with proC promoter in this continuous evolution experiment should generate pIII transcript levels comparable to those in part **(i)** while maintaining a lower number of $AP2_{H3}$ to ameliorate AP/SP recombination. Further reducing promoter strength to proA (t = 50 h) resulted in M13-like recombinant phages by t = 70 h (indicated by *). Sequencing analysis revealed that recombination occurred in the rrnB terminator in $AP2_{H3}$, yielding a recombinant SP (RC in **b**) that retained the o-antiRBS_{H3-1} sequence yet additionally integrated the AP-borne gIII cassette, controlled by o-RBS_{H3}. **k)** To limit this recombination, two synthetic terminator sequences were evaluated against the rrnB T1 terminator [5] in $AP2_{H3}$ in phage enrichment assays. The T0 (21 imm) + BS7 dual terminator [6] was found to provide the highest enrichment of SP_{H3} titers, and this final architecture is referred to as AP3 (**Figure 1b**). **l)** Phage enrichment assays using $AP3_{H3}$ in S3489 cells using various constitutive promoters. Data reflects the mean and standard deviation of 1–8 biological replicates.

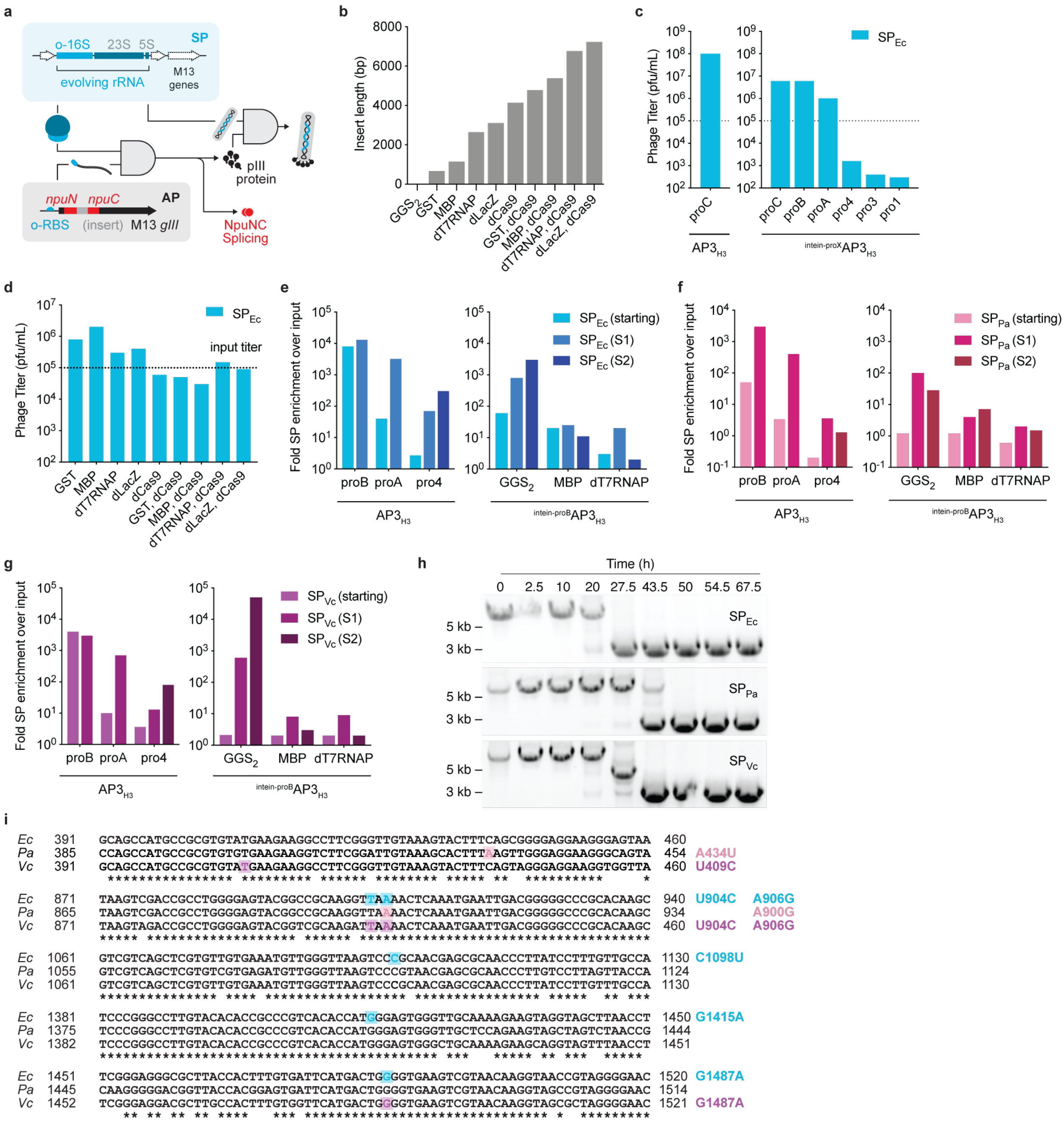

Supplementary Figure 3 | Design and validation of a split-intein pIII AP for continuous directed evolution. a) Schematic representation of a split-intein PACE selection. Functional orthogonal ribosomes encoded by the SP must efficiently translate an intein-*gIII* transcript from the accessory plasmid (AP), which undergoes trans-splicing to produce functional pIII. **b)** Genes used as the "insert" in part **(a)**. In all cases, prefix "d" indicates that the enzymatic activity has been inactivated by substitution at a catalytic residue. **c)** Overnight enrichment assays of $SP_{H3\text{-}1}$ in S3489 using AP_{H3} vs. $^{intein}AP_{H3}$ with a GSS_2 insert and driven by promoters of varying strengths. **d)** Phage enrichment assays of $SP_{H3\text{-}1}$ in S3489 using $^{intein\text{-}proB}AP3_{H3}$ and insertions of various lengths. **e-g)** Comparison of phage enrichment assays of **(e)** SP_{Ec}, **(f)** SP_{Pa}, and **(g)** SP_{Vc} starting sequences and after S1 and S2 of directed evolution in S3489 with $AP3_{H3}$ and $^{intein}AP_{H3}$ variants. **h)** O-rRNA operons encoded in the SPs underwent truncations after 27–43h of PACE, as shown by PCR products from amplifications of the entire rRNA operons throughout segment 1 (0–68 h). Data represents a single sample taken at each indicated timepoint for each

starting species (n = 1). PCR amplicons were generated using universal primers AB1792 (5'-actccttattacgcagtatgttagcaaac-3') and AB1793 (5'- taatggaaacttcctcatgaaaaagtctttag-3'). Interestingly, loss of the 23S subunit in SP_{Pa} and SP_{Vc} occurred concurrently with truncation of the 5S subunit while SP_{Ec} retained its 5S subunit throughout the first segment. At the end of the segments 3 and 4, several clones of SP_{Ec} underwent further truncation of the remaining 5S subunit. The truncated o-RNAs in all SP populations retained the 16S rRNA 3' processing sequence, highlighting its important role in base-paring with the 16S rRNA 5' leader sequence [7]. A similar rRNA truncation point was found in prior attempts to discover a minimal o-rRNA [8]. **I)** Pairwise sequence alignment of partial 16S rRNAs from *E. coli*, *P. aeruginosa*, and *V. cholerae*, noting consensus mutations in individual organisms and positions that were mutated independently in multiple organisms.

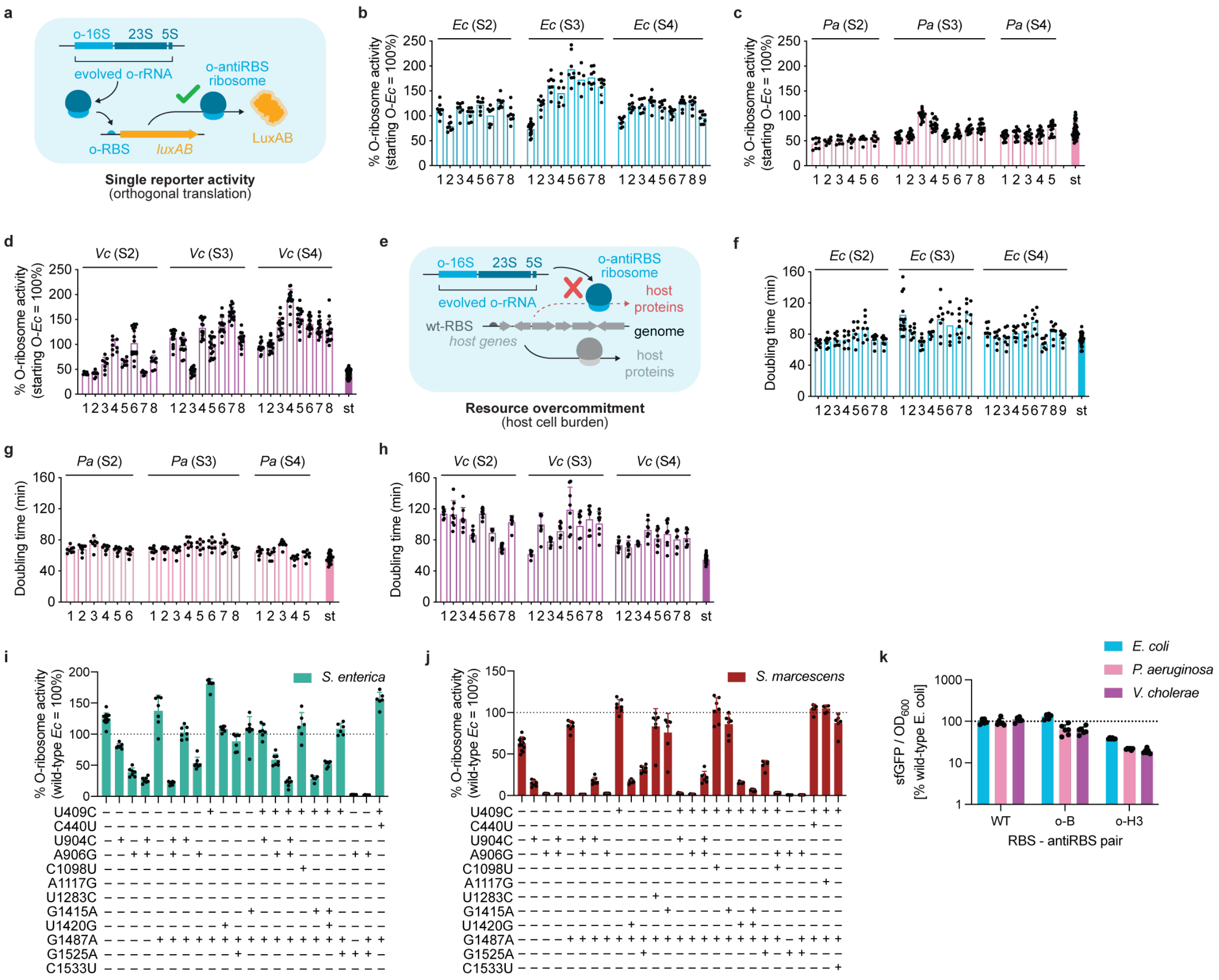

Supplementary Figure 4 | Analysis of evolved o-rRNA activities and transplantation of consensus mutations in heterologous o-rRNAs. a) Luminescence assays: upon induction of the o-rRNA EP, o-ribosomes are produced and translate an orthogonal luxAB transcript to produce luminescence. **b-d)** Evaluating oRibo-PACE-evolved *E. coli* **(b)**, *P. aeruginosa* **(c)**, or V. cholerae **(d)** o-rRNAs using an orthogonal luciferase reporter. For all luminescence assays, o-ribosome activities are expressed as % of starting *E. coli* o-ribosome. Data reflects mean and standard deviation of 8-72 biological replicates (n = 8-72). **e)** Cell burden: upon induction of the EP, cellular resources are diverted to the production of o-ribosomes, which are devoted to translation of the orthogonal luciferase transcript and cannot produce host proteins essential for host survival. Consequently, induction of o-ribosome production exerts a metabolic burden on the *E. coli* host [9], as manifested by changes in its doubling time. **f-h)** Quantifying the burden of oRibo-PACE-evolved *E. coli* **(f)**, *P. aeruginosa* **(g)**, or V. cholerae **(h)** o-rRNAs on S3489 cell doubling time. In all cases, the starting variant (st) is provided for comparison. Data reflects mean and standard deviation of 8-40 biological replicates (n = 8-40). **i-j)** Through analysis of consensus mutations discovered through oRibo-PACE, 12 mutations were transplanted into unrelated heterologous o-rRNAs from *Salmonella enterica* **(i)** and *Serratia marcescens* **(j)** and investigated using the orthogonal luciferase reporter. The combination of the two mutations U409C and G1487A showed the greatest improvement in both o-rRNAs. Data reflects mean and standard deviation of 6-14 biological replicates (n = 6-14). **k)** Comparison of sfGFP expression by ribosomes with cognate RBS-antiRBS pairs; wild-type (WT), oRBS-B (o-B) and oRBS-H3 (o-H3). Data reflect the mean and standard deviation of 6 biological replicates (n = 6).

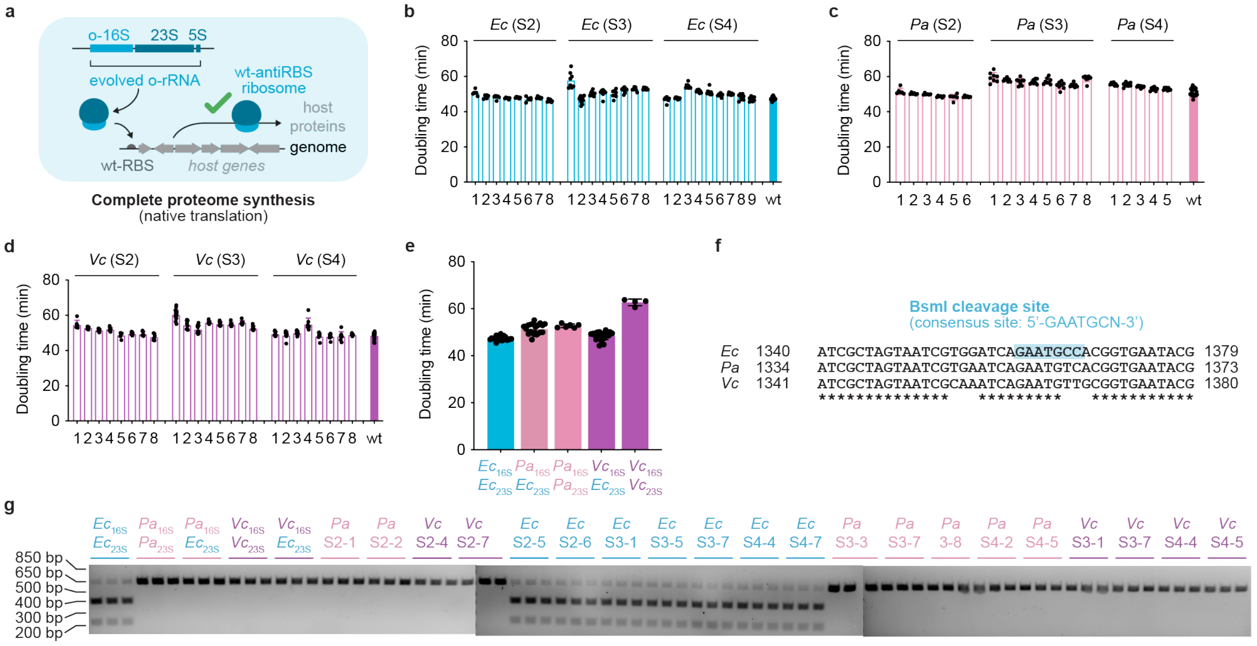

Supplementary Figure 5 | Complementation of SQ171 cells using evolved rRNAs. a) SQ171 complementation assays: evolved rRNA variants are engineered to encode wt-antiRBS to translate the cellular proteome necessary for the survival of the SQ171 host cells. B-D) Evaluating oRibo-PACE-evolved *E. coli* **(b)**, *P. aeruginosa* **(c)**, or V. cholerae **(d)** rRNAs using complemented SQ171 cell doubling time. In all cases, the starting variant bearing the wild-type antiRBS (wt) is provided for comparison. **e)** Comparison of complemented SQ171 strain doubling time using cognate 23S rRNA vs. *E. coli*-derived 23S rRNAs. Use of the *E. coli* 23S shows improved doubling time using both *P. aeruginosa* and V. cholerae 16S rRNAs, in agreement with the SP truncations during oRibo-PACE. Data reflects mean and standard deviation of 4-40 biological replicates (n = 4-40). All data presented in this report therefore implement the *E. coli* 23S rRNA alongside *P. aeruginosa* and V. cholerae 16S rRNAs. **f-g)** Complemented SQ171 strains encoding starting and evolved rRNAs in biological triplicate were PCR amplified using universal primers AB5606 (5'-cggtggagcatgtggttt-3') and AB5113 (5'-acgccttgcttttcactttc-3') to yield a ~668 bp PCR product. This PCR product is then digested using BsmI (New England Biolabs), which effectively identifies the rRNAs in SQ171 cells by either yielding two fragments (428 bp and 240 bp) to indicate an *E. coli* rRNA or no cleavage to indicate a heterologous rRNA. This analysis confirms correct rRNA plasmid exchange in all benchmarked SQ171 cells. Data reflects a single sample for each clone tested representing multiple samples from each oRibo-PACE selection regime (n = 1).

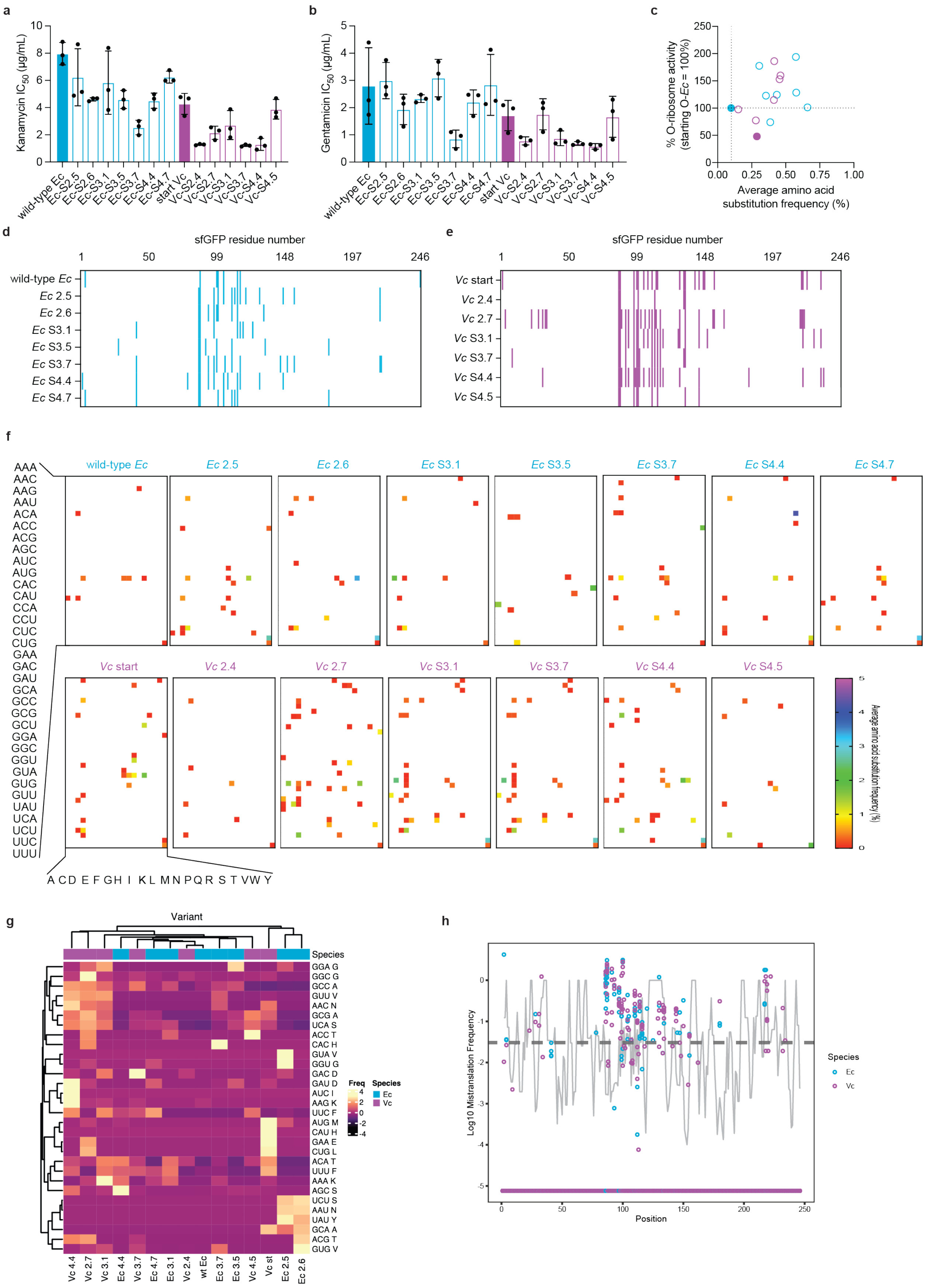
a
Kanamycin IC50 (µg/mL)
wild-type Ec
Ec-S2.5
Ec-S2.6
Ec-S3.1
Ec-S3.5
Ec-S3.7
Ec-S4.4
Ec-S4.7
start Vc
Vc-S2.4
Vc-S2.7
Vc-S3.1
Vc-S3.7
Vc-S4.4
Vc-S4.5
b
Gentamicin IC50 (µg/mL)
c
% O-ribosome activity (starting O-Ec = 100%)
Average amino acid substitution frequency (%)
d
sfGFP residue number
1 50 99 148 197 246
wild-type Ec
Ec 2.5
Ec 2.6
Ec S3.1
Ec S3.5
Ec S3.7
Ec S4.4
Ec S4.7
e
sfGFP residue number
Vc start
Vc 2.4
Vc 2.7
Vc S3.1
Vc S3.7
Vc S4.4
Vc S4.5
f
AAA AAC AAG AAU ACA ACC ACG AGC AUC AUG CAC CAU CCA CCU CUC CUG GAA GAC GAU GCA GCC GCG GCU GGA GGC GGU GUA GUG GUU UAU UCA UCU UUC UUU
A C D E F G H I K L M N P Q R S T V W Y
Average amino acid substitution frequency (%)
g
Variant
Species
GGA G
GGC G
GCC A
GUU V
AAC N
GCG A
UCA S
ACC T
CAC H
GUA V
GGU G
GAC D
GAU E
AUC I
AAG K
UUC F
AUG M
CAU H
GAA E
CUG L
ACA T
UUU F
AAA K
AGC S
UCU S
AAU N
UAU Y
GCA A
ACG T
GUG V
Freq
Species
Ec
Vc
Vc 4.4
Vc 2.7
Vc 3.1
Ec 4.4
Vc 3.7
Ec 4.7
Ec 3.1
Vc 2.4
wt Ec
Ec 3.7
Ec 3.5
Vc 4.5
Vc st
Ec 2.5
Ec 2.6
h
Log10 Mistranslation Frequency
Position
Species
Ec
Vc

Supplementary Figure 6 | Analysis of mistranslation SQ171 cells complemented with kinetically-enhanced rRNAs. IC_{50} values for the error-inducing aminoglycosides kanamycin **(a)** and gentamicin **(b)** for select *E. coli* and *V. cholerae* rRNA-complemented SQ171 strains. Data reflects the mean and standard deviation of 3 biological replicates run on different days (n = 3). **c)** Following sfGFP analysis for misincorporation via protein purification and LC-MS/MS analysis, we investigated correlation between kinetic o-ribosome translation activity and average amino acid substitution frequency. O-ribosome activity normalized to starting E. coli o-rRNA activity. Amino acid substitution frequency is calculated as the (%) substation abundance of sum of all peptides mapping to a specific residue in sfGFP. Data is shown as the mean and standard deviation of 3 biological replicates. The sites within sfGFP where substitutions were detected are shown for tested *E. coli*- **(d)** and *V. cholerae*-derived **(e)** rRNAs in SQ171 strains. Each row corresponds to a unique strain and columns to individual residues of sfGFP (1-246 residues). **f)** Comparison of observed amino acid substitutions and mRNA codon identities for select *E. coli* and *V. cholerae*-derived rRNAs in SQ171 strains. Color indicates (%) substitutions at a codon by a non-cognate amino acid. Each heatmap is labeled with the strain of *E. coli*-derived (top) or *V. cholerae*-derived (bottom) rRNA variant. **g)** Aggregated amino acid mis-incorporation for all select rRNA variants. **h)** Codon adaptation index of sfGFP for wild-type and evolved Ec and Vc variants.

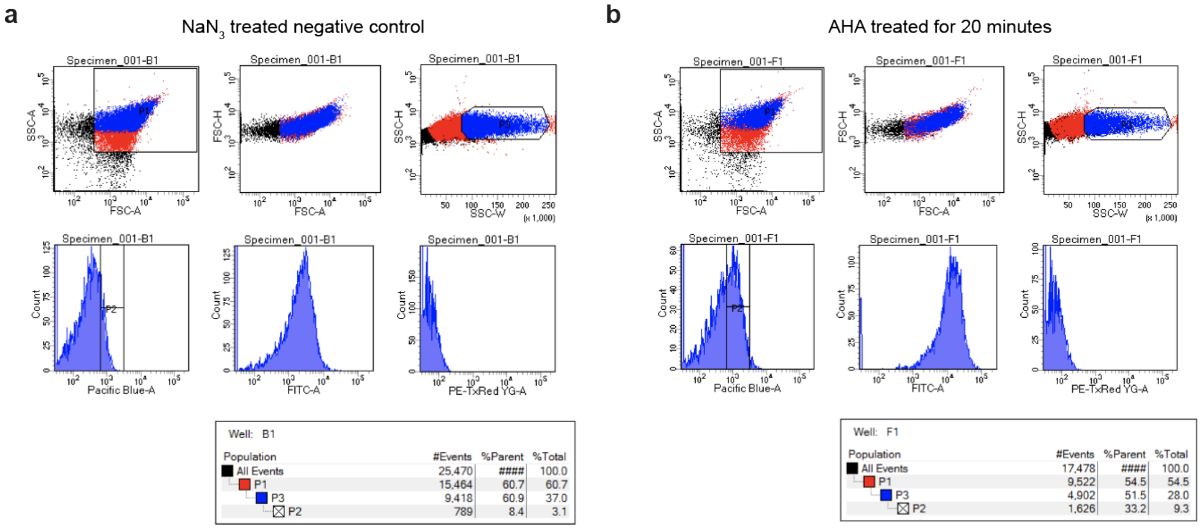

Supplementary Figure 7 | Gating strategy for AHA and Vitality. Cells were gated based on size first by (FSC-A x SSC-A) (P1) and then to discriminate singlets (SSC-W x SSC-H) (P3). FInally cells were gated based on AHA incorporation (Pacific-Blue-A) (P2). AHA staining was visualized as a histogram for (Pacifi-Blue-A) for NaN3 treated negative control **(a)** and positive sample labeled with AHA for 20 minutes **(b)**. Vitality staining was monitored by visualizing a histogram for (FITC-A) and (PE-TxRed YG-A).

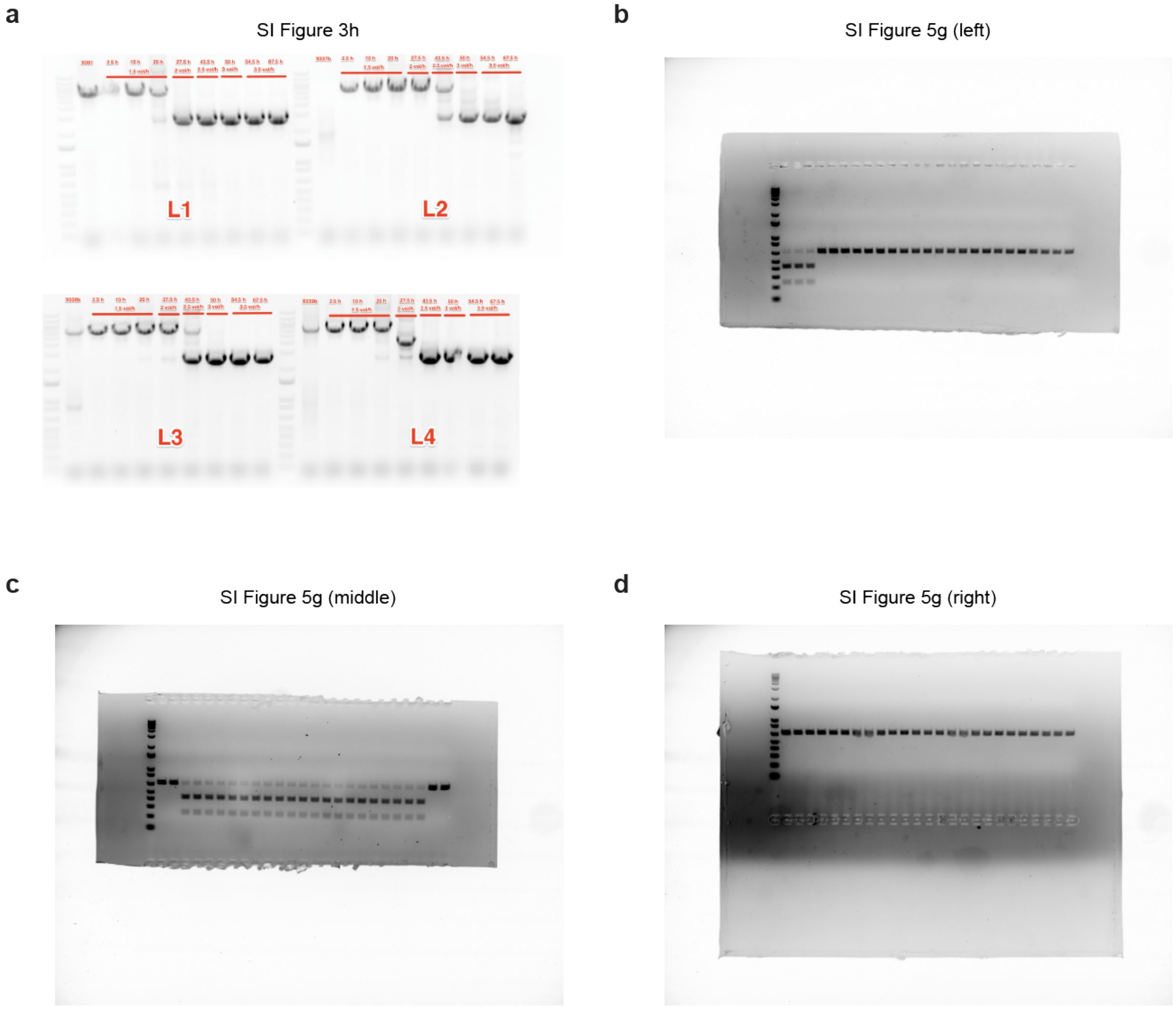

Supplementary Figure 8 | Unprocessed gel images. Unprocessed gel images relating to (**Supplementary Figure 3h**) **(a)** and (**Supplementary Figure 5g**) **(b-d)**.

PACE Segment	SP Plaque Number	% o-rRNA Activity (st o-Ec = 100%)[1]	SQ171 Doubling Time (min)[2]	Host Doubling Time (min)[3]	Residue Number															*E. coli* rRNA																	
					50	83	93	209	210	306	381	396	412	492	500	602	657	718	721	852	853	904	906	1015	1098	1189	1214	1258	1275	1415	1419	1445	1451	1461	1471	1487	1510
	st (o-antiRBS$_{H3-1}$)	100	N/A	73.1 ± 7.6	A	C	U	U	C	A	C	C	A	C	G	A	U	A	G	G	C	U	A	G	C	U	C	G	A	G	G	U	U	G	U	G	C
	wt (wt-antiRBS)	1.3 ± 0.4	47.5 ± 0.9	46.4 ± 3.6	A	C	U	U	C	A	C	C	A	C	G	A	U	A	G	G	C	U	A	G	C	U	C	G	A	G	G	U	U	G	U	G	C
1	1	N/A	N/A	N/A	**G**	C	U	U	C	A	C	C	A	C	G	A	U	**C**	G	G	C	U	A	G	C	U	C	G	A	G	G	U	U	G	U	G	C
1	2	N/A	N/A	N/A	A	C	U	U	C	A	C	C	A	**A**	G	A	U	A	G	G	C	U	A	G	C	U	C	G	A	G	G	U	U	G	U	G	C
1	3	N/A	N/A	N/A	A	C	U	U	C	A	C	C	A	C	G	A	U	A	G	G	C	U	A	G	C	U	C	G	A	G	G	U	U	G	U	G	C
1	4	N/A	N/A	N/A	A	C	**C**	U	C	A	C	C	A	C	G	A	U	A	G	G	C	U	**G**	G	C	U	C	G	A	G	G	U	U	G	U	G	C
2	1	112.1 ± 20.1	50.8 ± 1.5	67.9 ± 4.8	A	C	U	**G**	C	A	C	C	A	C	G	A	U	A	G	G	C	U	**G**	G	C	U	C	**A**	A	**A**	G	U	U	G	U	G	C
2	2	79.5 ± 14.3	48.6 ± 1	71 ± 7.1	A	C	U	U	C	A	C	C	A	C	G	A	U	A	G	G	C	U	A	G	C	U	C	G	A	**A**	G	U	U	G	U	G	C
2	3	114.1 ± 20	48.2 ± 1.1	73 ± 7.3	A	C	U	U	C	A	C	C	A	C	G	A	U	A	G	G	C	U	A	G	C	U	C	G	A	**A**	G	U	U	**U**	U	G	C
2	4	103.5 ± 17.8	47.6 ± 0.4	73.5 ± 9.1	A	C	U	U	C	A	C	C	A	C	G	A	U	A	**A**	G	C	U	A	G	C	U	C	G	A	**A**	G	U	**A**	G	U	G	C
2	5	122.8 ± 20.3	48 ± 0.4	81.5 ± 12.2	A	C	U	U	C	A	C	C	A	C	G	A	U	A	G	G	C	U	**G**	G	C	U	C	G	A	**A**	G	U	U	G	U	G	C
2	6	101.3 ± 21.8	47.2 ± 1.2	88 ± 14.6	A	C	U	U	C	A	C	C	A	C	G	A	U	A	G	G	C	U	A	G	C	U	C	G	A	**A**	G	U	U	G	U	**A**	C
2	7	127.4 ± 19.8	47.9 ± 0.6	73.2 ± 7.1	A	C	U	U	**–**	A	C	C	**G**	C	G	A	U	A	G	G	C	U	A	G	C	U	C	G	A	**A**	G	U	U	G	U	G	C
2	8	103.1 ± 21.7	46.2 ± 0.8	71.1 ± 6.3	A	C	U	U	C	A	C	C	A	C	G	A	U	A	G	G	C	U	A	G	C	**A**	C	G	A	**A**	G	U	U	G	U	G	C
3	1	74.1 ± 12.0	57.9 ± 4.5	105.2 ± 26.7	A	C	U	U	C	A	C	C	A	C	G	A	U	A	G	G	C	**C**	A	**A**	C	U	**A**	G	A	**A**	G	U	U	G	U	**A**	C
3	2	122.9 ± 17.9	46.9 ± 1.8	83.3 ± 7.2	A	C	U	U	C	A	C	C	A	C	G	A	U	A	G	G	C	U	A	G	C	U	C	G	A	**A**	G	U	U	G	U	G	C
3	3	162.0 ± 23.8	50.4 ± 1.4	69 ± 7.3	A	C	U	U	C	A	C	C	A	C	G	A	**C**	A	G	G	C	**C**	A	G	C	U	C	G	A	**A**	G	U	U	G	U	G	C
3	4	146.2 ± 27.2	50.7 ± 2.1	84.2 ± 8.7	A	C	U	U	C	A	C	C	A	C	G	A	U	A	G	G	**U**	U	**G**	G	C	U	C	G	A	G	G	U	U	G	U	**A**	C
3	5	193.9 ± 34.3	50.1 ± 2.2	99.7 ± 21.5	A	C	U	U	C	A	C	C	**C**	C	G	A	U	A	G	**A**	C	**C**	A	G	C	U	C	G	A	**A**	**A**	U	U	G	U	G	C
3	6	173.2 ± 27.4	52.2 ± 1.5	91.6 ± 23.2	A	C	U	U	C	A	C	C	A	C	G	A	U	A	G	G	C	U	**G**	G	**U**	U	C	G	A	G	G	U	**C**	G	U	**A**	C
3	7	177.5 ± 23.4	52.8 ± 1	88.9 ± 15.2	A	C	U	U	C	A	C	**U**	A	C	G	A	U	A	G	G	C	U	**G**	G	**U**	U	C	G	A	G	G	U	U	G	U	**A**	C
3	8 (x2)	159.1 ± 22.8	53 ± 0.6	105.8 ± 16.8	A	C	U	U	C	A	C	C	A	C	G	A	U	A	G	G	C	U	**G**	G	**U**	U	C	G	A	G	G	U	U	G	U	**A**	C
4	1	88.5 ± 11.5	47 ± 1.4	82.9 ± 10	A	C	U	U	C	A	C	C	A	C	G	A	U	A	G	G	C	U	A	G	C	U	C	G	A	G	G	U	U	G	U	G	C
4	2	119.5 ± 14.8	47.6 ± 0.5	75.7 ± 8.7	A	C	U	U	C	A	C	C	A	C	G	**G**	U	A	G	G	C	U	A	G	C	U	C	G	A	G	G	U	U	**A**	U	G	C
4	3	120.7 ± 16.2	54.5 ± 1.4	75.2 ± 11	A	C	**C**	U	C	A	**A**	C	A	C	G	A	U	A	G	G	C	U	A	G	C	U	C	G	A	G	G	U	U	G	U	G	C
4	4	128.5 ± 19.3	51.6 ± 0.8	81.9 ± 7.7	A	C	U	U	C	**G**	C	C	A	C	**A**	A	U	A	G	G	C	U	**G**	G	C	U	C	G	A	G	G	U	U	G	U	G	C
4	5	118.6 ± 16.6	51.2 ± 1.8	84.5 ± 12.6	A	**U**	U	U	C	A	C	C	A	C	G	A	U	A	G	G	C	U	A	G	C	U	C	G	A	G	G	U	U	G	U	G	**U**
4	6	107.1 ± 13.4	49.5 ± 0.9	96.3 ± 14	A	C	U	U	C	A	C	C	A	C	G	A	U	A	G	G	C	U	A	G	C	U	C	G	A	G	G	U	U	G	**C**	G	C
4	7	124.5 ± 16.1	50.1 ± 0.6	68 ± 7.4	A	C	U	U	C	A	C	C	A	C	G	A	U	A	G	G	C	U	A	G	C	U	C	G	**G**	G	G	U	U	G	U	G	C
4	8	124.5 ± 17.5	48.2 ± 1.7	84 ± 10.3	A	C	U	U	C	A	C	C	A	C	G	A	U	A	G	G	C	U	A	G	C	U	C	G	A	G	G	**C**	U	G	U	G	C
4	9	96.6 ± 13.8	47.3 ± 1.2	75.9 ± 7.2	A	C	U	U	C	A	C	C	A	C	G	A	U	A	G	G	C	U	A	G	C	U	C	G	A	G	G	U	U	G	U	G	C

Supplementary Table 1 | Sanger sequencing analysis of SP$_{Ec}$ samples during four separate segments of PACE. Mutations are colored on the basis of the stage in which they first became fixed in the evolving o-rRNA pool. Numbers in parentheses indicate the number of independent plaques isolated that carry the identical mutation(s). [1] % o-rRNA activity of each SP mutant is presented in bar graph form in **Supplementary Figure 3b**. [2] The doubling times (in mins) of SQ171 cells carrying EPs encoding evolved rRNA variants are presented in bar graph form in **Supplementary Figure 4b**. [3] The doubling times (in mins) of *E. coli* host cells carrying EPs encoding evolved rRNA variants are presented in bar graph form in **Supplementary Figure 3f**.

PACE Segment	SP Plaque Number	% o-rRNA Activity (st o-Ec = 100%)[1]	SQ171 Doubling Time (min)[2]	Host Doubling Time (min)[3]		*P. aeruginosa* rRNA																								
					Pa	83	187	230	415	434	449	555	606	644	833	900	984	1110	1128	1131	1139	1145	1247	1251	1268	1437	1453	1459	1472	1481
					Ec	86	193	236	421	440	455	561	612	650	839	906	990	1116	1134	1137	1145	1151	1253	1257	1274	1443	1459	1465	1478	1487
	st (o-antiRBS$_{H3-1}$)	70.7 ± 16.5	N/A	56.3 ± 5.8		G	A	A	U	A	G	U	C	G	U	A	C	U	C	C	C	A	G	U	G	U	A	A	U	G
cognate 50S	wt (wt-antiRBS)	N/A	52.7 ± 0.8	N/A		G	A	A	U	A	G	U	C	G	U	A	C	U	C	C	C	A	G	U	G	U	A	A	U	G
Ec 50S	wt (wt-antiRBS)	N/A	49.2 ± 1.2	N/A		G	A	A	U	A	G	U	C	G	U	A	C	U	C	C	C	A	G	U	G	U	A	A	U	G
1	1 (x2)	N/A	N/A	N/A		G	A	A	U	A	G	U	C	G	U	A	C	U	C	C	C	A	G	U	G	U	A	A	U	G
1	2	N/A	N/A	N/A		G	A	A	U	U	G	U	C	G	C	A	C	U	C	C	C	A	G	U	G	U	A	A	U	G
1	3*	N/A	N/A	N/A		G	A	A	U	U	G	U	C	G	U	A	C	U	C	C	C	A	G	U	G	U	A	A	U	G
1	5	N/A	N/A	N/A		G	A	A	U	A	A	U	C	G	U	A	C	U	U	C	C	A	G	U	G	U	A	A	U	G
1	6	N/A	N/A	N/A		G	A	A	U	A	G	U	C	G	U	A	C	U	C	C	C	A	A	G	G	U	A	A	U	G
1	7	N/A	N/A	N/A		G	G	A	U	A	G	U	C	A	U	A	C	U	C	C	C	A	G	U	G	U	A	A	U	G
1	8	N/A	N/A	N/A		G	A	A	U	U	G	U	C	G	U	A	C	U	C	C	C	G	G	U	G	U	A	A	U	G
2	1	44.5 ± 10.1	51.6 ± 1.8	67.3 ± 4.1		A	A	A	U	U	G	U	C	G	U	A	C	U	C	C	C	A	G	U	G	U	A	A	U	G
2	2 (x2)	49 ± 5	50.3 ± 0.5	68.4 ± 5.7		G	A	A	U	U	G	U	C	G	U	A	C	U	C	C	C	A	G	U	G	U	G	A	U	G
2	3	50.4 ± 7.6	50 ± 0.5	75.2 ± 7.4		G	A	A	U	U	G	U	C	G	U	A	C	U	C	C	C	A	G	U	G	U	G	A	U	G
2	4 (x2)	50.1 ± 7.4	48.7 ± 0.5	69.5 ± 4.2		G	A	A	U	U	G	U	C	G	U	A	C	U	C	C	C	A	G	U	G	U	A	A	U	G
2	5	52.8 ± 6.7	48.3 ± 1.7	66.9 ± 4.3		G	A	A	U	U	G	U	C	G	U	A	C	C	C	C	C	A	G	U	G	U	A	A	U	G
2	6	53.9 ± 8.6	48.5 ± 0.5	64.8 ± 5.2		G	A	A	C	U	G	U	C	G	U	A	C	U	C	C	C	A	G	U	G	U	A	A	U	G
3	1	57.8 ± 8.1	59.6 ± 2.7	65.9 ± 5.1		G	A	A	U	U	G	U	C	G	U	A	C	U	C	C	C	A	G	U	–	U	A	A	U	G
3	2	60.6 ± 7.7	58.1 ± 0.9	66.8 ± 5.4		G	A	A	U	U	G	U	C	G	U	A	C	U	C	C	C	A	G	U	G	U	A	A	U	G
3	3	100.9 ± 9.6	57.1 ± 1.9	66.1 ± 5.8		G	A	A	U	U	G	U	U	G	U	A	C	U	C	C	C	A	G	U	G	U	G	A	C	G
3	4	82.8 ± 10.7	57.1 ± 1.4	74.5 ± 7.8		G	A	A	U	U	G	U	C	G	U	G	U	U	C	C	C	A	G	U	G	U	A	A	U	G
3	5	59.9 ± 6.1	57.4 ± 1.8	72 ± 6.5		G	A	A	U	U	G	U	C	G	U	A	C	U	C	C	C	A	G	U	G	U	A	C	U	G
3	6	66.4 ± 8.3	55 ± 2	73.6 ± 6.5		G	A	A	U	U	G	U	C	G	U	G	C	U	C	C	U	A	G	U	G	U	A	A	U	G
3	7	73.2 ± 7.7	55.1 ± 1.1	73.9 ± 8		G	A	A	U	U	G	U	C	G	U	G	C	U	C	C	C	A	G	U	G	U	G	A	U	G
3	8	76.2 ± 11	58.8 ± 1.8	65.7 ± 5.1		G	A	A	U	U	G	C	C	G	U	G	C	U	C	C	C	A	G	U	G	U	A	A	U	G
4	1	59.5 ± 7.9	55.7 ± 0.8	64.1 ± 5.2		G	A	A	U	U	G	U	C	G	U	A	C	U	C	C	C	A	G	U	G	G	A	A	U	G
4	2	62.6 ± 10.1	55.4 ± 1.2	61.8 ± 6.2		G	A	A	U	U	G	U	C	G	U	A	C	U	C	C	C	A	G	U	G	U	A	A	U	A
4	3	59.4 ± 9.4	54.2 ± 0.6	74.5 ± 4.7		G	A	G	U	U	G	U	C	G	U	A	C	U	C	C	C	A	G	U	G	U	A	A	U	G
4	4	63.9 ± 10.3	52.9 ± 1	56.1 ± 4.4		G	A	A	U	U	G	U	C	G	U	A	C	U	C	U	C	A	G	U	G	U	A	A	U	G
4	5	74.5 ± 12.2	52.9 ± 0.7	60.7 ± 5.5		G	A	A	U	U	G	U	C	G	U	A	C	U	C	C	C	A	G	U	G	U	G	A	U	G
4	6 (x3)*					G	A	A	U	U	G	U	C	G	U	A	C	U	C	C	C	A	G	U	G	U	A	A	U	G

Supplementary Table 2 | Sanger sequencing analysis of SP_{Pa} samples during four separate segments of PACE. Mutations are colored on the basis of the stage in which they first became fixed in the evolving o-rRNA pool. Numbers in parentheses indicate the number of independent plaques isolated that carry the identical mutation(s). [1] % o-rRNA activity of each SP mutant is presented in bar graph form in **Supplementary Figure 3c**. [2] The doubling times (in mins) of SQ171 cells carrying EPs encoding evolved rRNA variants are presented in bar graph form in **Supplementary Figure 4c**. [3] The doubling times (in mins) of *E. coli* host cells carrying EPs encoding evolved rRNA variants are presented in bar graph form in **Supplementary Figure 3g**.

PACE Segment	SP Plaque Number	% o-rRNA Activity (st o-Ec = 100%)[1]	SQ171 Doubling Time (min)[2]	Host Doubling Time (min)[3]
	st (o-antiRBS$_{H3-1}$)	41.6 ± 7.6	N/A	55.2 ± 4.2
cognate 50S	wt (wt-antiRBS)	N/A	62.7 ± 1.4	N/A
Ec 50S	wt (wt-antiRBS)	N/A	47.6 ± 2.6	N/A
1	1	N/A	N/A	N/A
1	2	N/A	N/A	N/A
1	3	N/A	N/A	N/A
1	4	N/A	N/A	N/A
1	5	N/A	N/A	N/A
1	6	N/A	N/A	N/A
1	7	N/A	N/A	N/A
1	8	N/A	N/A	N/A
2	1	42.1 ± 2.8	54.8 ± 2.5	114.2 ± 5.9
2	2	40.8 ± 5.8	52.9 ± 0.7	113.2 ± 17.5
2	3	61 ± 13.1	51.5 ± 0.9	107.5 ± 14.2
2	4	97.6 ± 13.3	52.1 ± 1.1	86.9 ± 6.0
2	5	64 ± 7.4	48.8 ± 1.6	114.4 ± 6.0
2	6	103.3 ± 29.1	49.7 ± 0.8	89.5 ± 5.7
2	7	42.7 ± 4	49.4 ± 1.3	70.3 ± 4.3
2	8	65.4 ± 12.7	47.8 ± 1.8	103.1 ± 8.3
3	1	114 ± 15.3	60.4 ± 2.8	62.3 ± 4.4
3	2	98.9 ± 15.8	54.6 ± 2.4	100.2 ± 14.7
3	3	46.6 ± 7.3	52.1 ± 2.2	78.5 ± 5.0
3	4	133.8 ± 18	56.0 ± 0.8	91.6 ± 11.2
3	5	95.1 ± 18.8	55.2 ± 1.2	119.2 ± 28.6
3	6	137 ± 17.1	54.9 ± 1.1	98.3 ± 17.9
3	7	161.7 ± 13.2	55.9 ± 1.1	107.2 ± 16.9
3	8	110 ± 13.7	52.7 ± 1.2	101.5 ± 15.4
4	1	93.9 ± 9.8	49.5 ± 1.3	73.8 ± 6.6
4	2	97.4 ± 12.2	48.7 ± 2.1	70.8 ± 8.2
4	3	135.2 ± 18.5	50.0 ± 1.2	75.1 ± 2.2
4	4	190.6 ± 19.7	54.9 ± 3.5	93.2 ± 11.9
4	5	153.1 ± 19.8	48.2 ± 1.8	82.2 ± 12.1
4	6	141.3 ± 14.7	47.9 ± 1.5	88.1 ± 17.0
4	7	132.9 ± 15.6	48.2 ± 2.5	81.0 ± 11.9
4	8	127.6 ± 22.5	49.0 ± 0.5	82.8 ± 10.4

V. cholerae rRNA

PACE Segment	SP Plaque Number	Vc	4	50	70	92	149	164	234	333	349	409	412	426	454	543	627	631	650	691	694	846	848	856	904	906	965
		Ec	4	50	70	92	149	164	234	333	349	409	412	426	454	543	627	631	650	691	694	846	848	856	904	906	965
	st (o-antiRBS$_{H3-1}$)		U	A	C	U	A	G	C	U	A	U	A	U	G	–	–	U	–	G	A	G	C	U	U	A	U
cognate 50S	wt (wt-antiRBS)		U	A	C	U	A	G	C	U	A	U	A	U	G	–	–	U	–	G	A	G	C	U	U	A	U
Ec 50S	wt (wt-antiRBS)		U	A	C	U	A	G	C	U	A	U	A	U	G	–	–	U	–	G	A	G	C	U	U	A	U
1	1		U	A	C	U	A	G	C	U	A	U	A	U	G	–	–	U	–	G	A	G	C	U	**C**	A	U
1	2		U	A	C	U	A	G	C	U	A	U	A	U	G	–	–	U	–	G	A	G	C	U	U	**G**	**C**
1	3		U	A	C	U	A	G	C	U	A	U	A	U	G	**G**	–	U	–	G	A	G	C	U	**C**	A	U
1	4		U	A	C	U	A	G	C	U	A	U	A	U	G	–	–	U	–	G	A	G	C	U	**C**	A	U
1	5		U	A	C	**C**	A	G	C	U	A	U	A	U	G	–	–	U	–	G	A	G	C	U	**C**	A	U
1	6		**C**	A	C	U	A	G	C	U	A	U	A	U	G	–	**G**	U	–	G	A	G	C	U	U	**G**	U
1	7		U	A	C	U	A	G	C	U	A	U	**C**	U	G	–	–	U	–	G	A	G	C	U	**C**	A	U
1	8		U	A	**U**	U	A	G	C	U	A	U	A	U	G	–	–	U	–	**A**	A	G	C	U	**C**	A	U
2	1		U	A	C	U	A	G	C	U	A	U	A	U	G	–	–	**C**	–	G	A	G	C	U	**C**	**G**	U
2	2		U	A	C	U	A	G	**U**	U	A	U	A	U	G	–	–	U	–	G	A	G	C	U	**C**	**G**	U
2	3		U	A	C	U	A	G	C	U	A	U	A	U	**A**	–	–	U	–	G	A	G	C	U	**C**	**G**	U
2	4		U	A	C	U	A	G	C	U	A	U	A	U	G	–	–	U	–	G	A	G	C	U	**C**	**G**	U
2	5		U	A	C	U	A	G	C	U	A	U	A	U	G	–	–	U	–	G	A	G	C	U	**C**	**G**	U
2	6		U	A	C	U	A	G	C	U	A	U	A	U	G	–	–	U	–	G	**G**	G	C	U	**C**	**G**	U
2	7		U	A	C	U	A	G	C	U	A	U	A	U	G	–	–	U	–	G	A	G	C	U	**C**	**G**	U
2	8		U	A	C	U	A	G	C	U	A	U	A	U	G	–	–	U	–	G	A	G	C	U	**C**	**G**	U
3	1		U	A	C	U	A	G	C	U	A	U	A	U	G	–	–	U	**G**	G	A	G	**U**	U	**C**	A	U
3	2		U	A	C	U	A	G	C	U	A	**C**	A	U	G	–	–	U	–	G	A	G	C	U	**C**	**G**	U
3	3		U	**G**	C	U	A	G	C	U	**G**	U	A	U	G	–	–	U	–	G	A	G	C	U	**C**	A	U
3	4		U	A	C	U	A	G	C	U	A	**C**	A	U	G	–	–	U	–	G	A	**U**	C	U	**C**	**G**	U
3	5		U	A	C	U	A	G	C	U	A	**C**	A	U	G	–	–	U	–	G	A	G	C	U	**C**	**G**	U
3	6		U	A	C	U	A	G	C	U	A	**C**	A	U	G	–	–	U	–	G	A	G	C	U	**C**	**G**	U
3	7		U	A	C	U	**G**	G	C	U	A	**C**	A	U	G	–	–	U	–	G	A	G	C	U	**C**	**G**	U
3	8		U	A	C	U	A	G	C	U	A	**C**	A	U	G	–	–	U	–	G	A	G	C	U	**C**	**G**	U
4	1		U	A	C	U	A	**A**	C	U	A	U	A	U	G	–	–	U	–	G	A	G	C	U	U	**G**	U
4	2		U	A	C	U	A	G	C	**G**	A	U	A	U	G	–	–	U	–	G	A	G	C	U	U	**G**	U
4	3		U	A	C	U	A	G	C	U	A	U	A	U	G	–	–	U	–	G	A	G	C	U	U	**G**	U
4	4		U	A	C	U	A	G	C	U	A	U	A	**C**	G	–	–	U	–	G	A	G	C	U	U	**G**	U
4	5		U	A	C	U	A	G	C	U	A	U	A	U	G	–	–	U	–	G	A	G	C	U	U	**G**	U
4	6		U	A	C	U	A	G	C	U	A	U	A	U	G	–	–	U	–	G	A	G	C	U	U	**G**	U
4	7		U	A	C	U	A	G	C	U	A	U	A	U	G	–	–	U	–	G	A	G	C	U	U	**G**	U
4	8		U	A	C	U	A	G	C	U	A	U	A	U	G	–	–	U	–	G	A	G	C	**C**	U	**G**	U

PACE Segment	SP Plaque Number	Vc	1005	1006	1100	1109	1117	1123	1137	1151	1167	1213	1280	1284	1300	1313	1420	1421	1443	1458	1460	1478	1479	1488	1512	1526	1534
		Ec	1005	1006	1100	1109	1117	1123	–	1150	1166	1212	1279	1283	1299	1312	1419	1420	1442	1457	1459	1477	1478	1487	1511	1525	1533
	st (o-antiRBS$_{H3-1}$)		A	U	C	C	A	G	A	C	G	U	A	U	A	G	G	U	G	G	A	–	U	G	C	C	C
cognate 50S	wt (wt-antiRBS)		A	U	C	C	A	G	A	C	G	U	A	U	A	G	G	U	G	G	A	–	U	G	C	C	C
Ec 50S	wt (wt-antiRBS)		A	U	C	C	A	G	A	C	G	U	A	U	A	G	G	U	G	G	A	–	U	G	C	C	C
1	1		A	U	C	C	A	G	A	C	G	U	A	U	A	G	G	U	G	**U**	A	–	U	G	C	C	C
1	2		A	U	C	C	A	G	A	C	G	U	A	U	A	G	G	U	G	G	A	–	U	G	C	C	C
1	3		A	U	C	C	A	G	A	C	G	U	A	U	A	G	G	U	G	G	A	–	U	**A**	C	C	**U**
1	4		A	U	C	C	A	G	A	C	G	U	**G**	U	A	G	G	U	G	G	A	–	U	G	C	C	C
1	5		A	U	C	C	A	G	A	C	G	U	A	U	A	G	G	U	G	G	A	–	U	**A**	C	C	C
1	6		A	U	C	C	A	**A**	A	C	G	U	A	U	A	G	G	U	G	G	A	–	U	G	C	C	C
1	7		A	U	**U**	C	A	G	A	C	G	U	A	U	A	G	G	U	G	G	A	–	U	**A**	C	C	C
1	8		A	U	C	C	A	G	A	C	G	U	A	U	A	G	G	U	G	G	A	–	U	**A**	C	C	C
2	1		A	U	C	C	A	G	A	C	G	U	A	U	A	G	G	**G**	G	G	A	–	U	G	C	C	C
2	2		A	U	C	C	A	G	A	C	G	U	A	**C**	A	G	G	**G**	G	G	A	–	U	G	C	C	C
2	3		A	U	C	C	A	G	A	C	G	U	A	U	A	G	G	**G**	G	G	A	–	U	G	C	C	C
2	4		A	U	C	C	A	G	A	C	G	U	A	U	A	G	G	**G**	G	G	A	**U**	U	G	C	C	C
2	5		A	U	C	C	A	G	A	C	G	U	A	**C**	A	G	G	**G**	G	G	A	–	U	G	C	C	C
2	6		A	U	C	C	A	G	A	C	G	U	A	**C**	A	G	G	**G**	G	G	A	–	U	G	C	C	C
2	7		A	U	C	C	A	G	A	C	G	**C**	A	U	A	G	G	U	G	G	A	–	U	**A**	C	C	C
2	8		A	U	C	C	A	G	A	C	G	U	A	**C**	A	G	G	U	**A**	G	A	–	U	G	C	C	C
3	1		A	U	C	C	**G**	G	A	C	G	U	A	**C**	A	G	G	U	G	G	A	–	U	**A**	C	C	**U**
3	2		A	U	C	C	A	G	A	C	G	U	A	U	A	G	G	U	G	G	A	–	U	**A**	C	C	C
3	3		A	U	C	C	**G**	G	A	C	**A**	U	A	**C**	A	G	G	U	G	G	A	–	U	**A**	C	C	**U**
3	4		A	U	C	C	A	G	A	C	G	U	A	U	A	G	G	U	G	G	A	–	U	**A**	C	C	C
3	5		A	U	C	C	A	G	A	C	G	U	A	U	A	G	G	U	G	G	A	–	U	**A**	C	C	C
3	6		A	U	C	C	A	G	A	C	G	U	A	U	A	**A**	G	U	G	G	A	–	U	**A**	**U**	C	C
3	7		A	U	C	**U**	A	G	A	C	G	U	A	U	A	G	G	**C**	G	G	A	–	U	**A**	C	C	C
3	8		A	U	C	C	A	G	A	**U**	G	U	A	U	**G**	G	G	U	G	G	A	–	U	**A**	C	C	C
4	1		**G**	U	C	C	A	G	A	C	G	U	A	U	A	G	G	U	G	G	A	–	U	G	C	**A**	C
4	2		A	U	**U**	C	A	G	A	C	G	U	A	U	A	G	G	U	G	G	A	–	U	G	C	**A**	C
4	3		A	U	C	C	A	G	A	C	G	U	A	U	A	G	**A**	U	G	G	A	–	U	G	C	**A**	C
4	4		A	U	C	C	A	G	A	C	G	U	A	U	A	G	G	U	G	G	**C**	–	U	**A**	C	**A**	C
4	5		A	U	C	C	A	G	**G**	C	G	U	A	U	A	G	G	U	G	G	A	–	U	G	C	**A**	C
4	6		A	U	C	C	A	G	A	C	G	U	A	U	A	G	G	U	G	G	A	–	U	G	C	**A**	C
4	7		A	**C**	C	C	A	G	A	C	G	U	A	U	A	G	G	U	G	G	A	–	**C**	G	C	**A**	C
4	8		A	U	C	C	A	G	A	C	G	U	A	**C**	A	G	G	U	G	G	A	–	U	G	C	**A**	C

Supplementary Table 3 | Sanger sequencing analysis of SP$_{Vc}$ samples during four separate segments of PACE. Mutations are colored on the basis of the stage in which they first became fixed in the evolving o-rRNA pool. Numbers in parentheses indicate the number of independent plaques isolated that carry the identical mutation(s). [1] % o-rRNA activity of each SP mutant is presented in bar graph form in **Supplementary Figure 3d**. [2] The doubling times (in mins) of SQ171 cells carrying EPs encoding evolved rRNA variants are presented in bar graph form in **Supplementary Figure 4d**. [3] The doubling times (in mins) of *E. coli* host cells carrying EPs encoding evolved rRNA variants are presented in bar graph form in **Supplementary Figure 3h**.

Strain	Description	Genotype	Reference
S1021	–	*F^{-} endA1 recA1 galE15 galK16 nupG rpsL ΔlacIZYA araD139 Δ(ara,leu)7697 mcrA Δ(mrr-hsdRMS-mcrBC) proBA::pir116 araE201 ΔrpoZ Δflu ΔcsgABCDEFG ΔpgaC λ–*	[10]
S2057	–	*F' proA^{+}B^{+} Δ(lacIZY) zzf::Tn10 lacI^{Q1} P$_{N25}$-tetR luxCDE P$_{psp}$(AR2) lacZ luxR P$_{lux}$ groESL / fhuA2 glnV Δ(lac-proAB) thi-1 Δ(hsdS-mcrB)5*	[11]
S2060	S1021 x S2057	*F' proA^{+}B^{+} Δ(lacIZY) zzf::Tn10 lacI^{Q1} P$_{N25}$-tetR luxCDE P$_{psp}$(AR2) lacZ luxR P$_{lux}$ groESL / endA1 recA1 galE15 galK16 nupG rpsL ΔlacIZYA araD139 Δ(ara,leu)7697 mcrA Δ(mrr-hsdRMS-mcrBC) proBA::pir116 araE201 ΔrpoZ Δflu ΔcsgABCDEFG ΔpgaC λ–*	[11]
S3300	S1021 Δ*hpf*	*F^{-} endA1 recA1 galE15 galK16 nupG rpsL ΔlacIZYA araD139 Δ(ara,leu)7697 mcrA Δ(mrr-hsdRMS-mcrBC) proBA::pir116 araE201 ΔrpoZ Δflu ΔcsgABCDEFG ΔpgaC Δhpf λ–*	This work
S3301	S1021 Δ*rmf*	*F^{-} endA1 recA1 galE15 galK16 nupG rpsL ΔlacIZYA araD139 Δ(ara,leu)7697 mcrA Δ(mrr-hsdRMS-mcrBC) proBA::pir116 araE201 ΔrpoZ Δflu ΔcsgABCDEFG ΔpgaC Δrmf λ–*	This work
S3302	S1021 Δ*raiA*	*F^{-} endA1 recA1 galE15 galK16 nupG rpsL ΔlacIZYA araD139 Δ(ara,leu)7697 mcrA Δ(mrr-hsdRMS-mcrBC) proBA::pir116 araE201 ΔrpoZ Δflu ΔcsgABCDEFG ΔpgaC ΔraiA λ–*	This work
S3303	S1021 Δ*rsfS*	*F^{-} endA1 recA1 galE15 galK16 nupG rpsL ΔlacIZYA araD139 Δ(ara,leu)7697 mcrA Δ(mrr-hsdRMS-mcrBC) proBA::pir116 araE201 ΔrpoZ Δflu ΔcsgABCDEFG ΔpgaC ΔrsfS λ–*	This work
S3314	S1021 Δ*hpf* Δ*raiA*	*F^{-} endA1 recA1 galE15 galK16 nupG rpsL ΔlacIZYA araD139 Δ(ara,leu)7697 mcrA Δ(mrr-hsdRMS-mcrBC) proBA::pir116 araE201 ΔrpoZ Δflu ΔcsgABCDEFG ΔpgaC Δhpf ΔraiA λ–*	This work
S3317	S3300 x S2057	*F' proA^{+}B^{+} Δ(lacIZY) zzf::Tn10 lacI^{Q1} P$_{N25}$-tetR luxCDE P$_{psp}$(AR2) lacZ luxR P$_{lux}$ groESL / endA1 recA1 galE15 galK16 nupG rpsL ΔlacIZYA araD139 Δ(ara,leu)7697 mcrA Δ(mrr-hsdRMS-mcrBC) proBA::pir116 araE201 ΔrpoZ Δflu ΔcsgABCDEFG ΔpgaC Δhpf λ–*	This work
S3318	S3301 x S2057	*F' proA^{+}B^{+} Δ(lacIZY) zzf::Tn10 lacI^{Q1} P$_{N25}$-tetR luxCDE P$_{psp}$(AR2) lacZ luxR P$_{lux}$ groESL / endA1 recA1 galE15 galK16 nupG rpsL ΔlacIZYA araD139 Δ(ara,leu)7697 mcrA Δ(mrr-hsdRMS-mcrBC) proBA::pir116 araE201 ΔrpoZ Δflu ΔcsgABCDEFG ΔpgaC Δrmf λ–*	This work
S3319	S3302 x S2057	*F' proA^{+}B^{+} Δ(lacIZY) zzf::Tn10 lacI^{Q1} P$_{N25}$-tetR luxCDE P$_{psp}$(AR2) lacZ luxR P$_{lux}$ groESL / endA1 recA1 galE15 galK16 nupG rpsL ΔlacIZYA araD139 Δ(ara,leu)7697 mcrA Δ(mrr-hsdRMS-mcrBC) proBA::pir116 araE201 ΔrpoZ Δflu ΔcsgABCDEFG ΔpgaC ΔraiA λ–*	This work
S3320	S3303 x S2057	*F' proA^{+}B^{+} Δ(lacIZY) zzf::Tn10 lacI^{Q1} P$_{N25}$-tetR luxCDE P$_{psp}$(AR2) lacZ luxR P$_{lux}$ groESL / endA1 recA1 galE15 galK16 nupG rpsL ΔlacIZYA araD139 Δ(ara,leu)7697 mcrA Δ(mrr-hsdRMS-mcrBC) proBA::pir116 araE201 ΔrpoZ Δflu ΔcsgABCDEFG ΔpgaC ΔrsfS λ–*	This work
S3322	S3314 x S2057	*F' proA^{+}B^{+} Δ(lacIZY) zzf::Tn10 lacI^{Q1} P$_{N25}$-tetR luxCDE P$_{psp}$(AR2) lacZ luxR P$_{lux}$ groESL / endA1 recA1 galE15 galK16 nupG rpsL ΔlacIZYA araD139 Δ(ara,leu)7697 mcrA Δ(mrr-hsdRMS-mcrBC) proBA::pir116 araE201 ΔrpoZ Δflu ΔcsgABCDEFG ΔpgaC Δhpf ΔraiA Δhpf λ–*	This work
S3485	S1021 Δ*hpf* Δ*fhuA*	*endA1 recA1 galE15 galK16 nupG rpsL ΔlacIZYA araD139 Δ(ara,leu)7697 mcrA Δ(mrr-hsdRMS-mcrBC) proBA::pir116 araE201 ΔrpoZ Δflu ΔcsgABCDEFG ΔpgaC Δhpf ΔfhuA λ–*	This work
S3489	S3485 x S2057	*F' proA^{+}B^{+} Δ(lacIZY) zzf::Tn10 lacI^{Q1} P$_{N25}$-tetR luxCDE P$_{psp}$(AR2) lacZ luxR P$_{lux}$ groESL / endA1 recA1 galE15 galK16 nupG rpsL ΔlacIZYA araD139 Δ(ara,leu)7697 mcrA Δ(mrr-hsdRMS-mcrBC) proBA::pir116 araE201 ΔrpoZ Δflu ΔcsgABCDEFG ΔpgaC Δhpf ΔfhuA λ–*	This work

Supplementary Table 4 | Genotypes of all bacterial strains used in this work.

Supplementary References

1 Peterson, J. & Phillips, G. J. New pSC101-derivative cloning vectors with elevated copy numbers. *Plasmid* **59**, 193-201, doi:10.1016/j.plasmid.2008.01.004 (2008).

2 Davis, J. H., Rubin, A. J. & Sauer, R. T. Design, construction and characterization of a set of insulated bacterial promoters. *Nucleic Acids Research* **39**, 1131-1141, doi:10.1093/nar/gkq810 (2011).

3 Killmann, H., Videnov, G., Jung, G., Schwarz, H. & Braun, V. Identification of receptor binding sites by competitive peptide mapping: phages T1, T5, and phi 80 and colicin M bind to the gating loop of FhuA. *Journal of Bacteriology* **177**, 694-698, doi:10.1128/jb.177.3.694-698.1995 (1995).

4 Badran, A. H. & Liu, D. R. Development of potent in vivo mutagenesis plasmids with broad mutational spectra. *Nat Commun* **6**, 8425, doi:10.1038/ncomms9425 (2015).

5 Reynolds, R., Bermúdez-Cruz, R. M. & Chamberlin, M. J. Parameters affecting transcription termination by Escherichia coli RNA polymerase. I. Analysis of 13 rho-independent terminators. *J Mol Biol* **224**, 31-51, doi:10.1016/0022-2836(92)90574-4 (1992).

6 McDowell, J. C., Roberts, J. W., Jin, D. J. & Gross, C. Determination of intrinsic transcription termination efficiency by RNA polymerase elongation rate. *Science* **266**, 822-825, doi:10.1126/science.7526463 (1994).

7 Brosius, J., Dull, T. J., Sleeter, D. D. & Noller, H. F. Gene organization and primary structure of a ribosomal RNA operon from Escherichia coli. *J Mol Biol* **148**, 107-127, doi:10.1016/0022-2836(81)90508-8 (1981).

8 An, W. & Chin, J. W. Synthesis of orthogonal transcription-translation networks. *Proceedings of the National Academy of Sciences* **106**, 8477-8482, doi:10.1073/pnas.0900267106 (2009).

9 Orelle, C. *et al.* Protein synthesis by ribosomes with tethered subunits. *Nature* **524**, 119-124, doi:10.1038/nature14862 (2015).

10 Carlson, J. C., Badran, A. H., Guggiana-Nilo, D. A. & Liu, D. R. Negative selection and stringency modulation in phage-assisted continuous evolution. *Nature Chemical Biology* **10**, 216-222, doi:10.1038/nchembio.1453 (2014).

11 Hubbard, B. P. *et al.* Continuous directed evolution of DNA-binding proteins to improve TALEN specificity. *Nature Methods* **12**, 939-942, doi:10.1038/nmeth.3515 (2015).
