# Supplementary material for: Directed evolution of rRNA improves translation kinetics and recombinant protein yield
Source: Nat Commun. 2021 Sep 24;12:5638. doi: 10.1038/s41467-021-25852-5 (PMC8463689; doi:10.1038/s41467-021-25852-5)
Supplement: Supplementary file 2 — Reporting Summary [file 41467_2021_25852_MOESM2_ESM.pdf]

nature portfolio

Corresponding author(s): Ahmed H. Badran

Last updated by author(s): Aug 19, 2021

Reporting Summary

Nature Portfolio wishes to improve the reproducibility of the work that we publish. This form provides structure for consistency and transparency in reporting. For further information on Nature Portfolio policies, see our Editorial Policies and the Editorial Policy Checklist.

Statistics

For all statistical analyses, confirm that the following items are present in the figure legend, table legend, main text, or Methods section.

n/a	Confirmed	
☐	☒	The exact sample size (*n*) for each experimental group/condition, given as a discrete number and unit of measurement
☐	☒	A statement on whether measurements were taken from distinct samples or whether the same sample was measured repeatedly
☒	☐	The statistical test(s) used AND whether they are one- or two-sided<br>*Only common tests should be described solely by name; describe more complex techniques in the Methods section.*
☒	☐	A description of all covariates tested
☐	☒	A description of any assumptions or corrections, such as tests of normality and adjustment for multiple comparisons
☐	☒	A full description of the statistical parameters including central tendency (e.g. means) or other basic estimates (e.g. regression coefficient) AND variation (e.g. standard deviation) or associated estimates of uncertainty (e.g. confidence intervals)
☒	☐	For null hypothesis testing, the test statistic (e.g. *F*, *t*, *r*) with confidence intervals, effect sizes, degrees of freedom and *P* value noted<br>*Give P values as exact values whenever suitable.*
☒	☐	For Bayesian analysis, information on the choice of priors and Markov chain Monte Carlo settings
☒	☐	For hierarchical and complex designs, identification of the appropriate level for tests and full reporting of outcomes
☒	☐	Estimates of effect sizes (e.g. Cohen's *d*, Pearson's *r*), indicating how they were calculated

Our web collection on statistics for biologists contains articles on many of the points above.

Software and code

Policy information about availability of computer code

Data collection	Plate reader data was generated using (Tecan SparkControl v2.3 and Molecular Devices SoftMax Pro v6.4). Flow cytometry data was generated using (BD Biosciences flow cytometer LSR II HTS with excitation lasers at 405, 488 and 561 nm and emission filters at 450/50, 515/20 and 610/20 nm). LC-MS/MS data was collected by reversed phase high performance liquid chromatography (HPLC) using Waters NanoAcquity pumps and autosampler and a Orbitrap Elite mass spectrometer (ThermoFisher) using a nano flow configuration.
Data analysis	GraphPad Prism (version 9.1.0) was used for plotting and data analysis, including calculation of means, standard deviations, and regression lines. The Growthcurver package (version 0.3.0) in R (version 3.5.2) was used to calculate doubling times from growth curves. Flow cytometry data was analyzed with (FlowJo v10). LC-MS/MS fragmentation spectra were correlated against custom databases using (PEAKS Studio vX+). Code used to identify codon adaptation index and amino acid mis-incorporation are available at (https://github.com/sbratulic/o-Ribo-PACE).

For manuscripts utilizing custom algorithms or software that are central to the research but not yet described in published literature, software must be made available to editors and reviewers. We strongly encourage code deposition in a community repository (e.g. GitHub). See the Nature Portfolio guidelines for submitting code & software for further information.

Data

Policy information about availability of data

All manuscripts must include a data availability statement. This statement should provide the following information, where applicable:

- Accession codes, unique identifiers, or web links for publicly available datasets
- A description of any restrictions on data availability
- For clinical datasets or third party data, please ensure that the statement adheres to our policy

All data generated or analysed during this study are included in this published article (and its supplementary information files).

Field-specific reporting

Please select the one below that is the best fit for your research. If you are not sure, read the appropriate sections before making your selection.

☒ Life sciences ☐ Behavioural & social sciences ☐ Ecological, evolutionary & environmental sciences

For a reference copy of the document with all sections, see nature.com/documents/nr-reporting-summary-flat.pdf

Life sciences study design

All studies must disclose on these points even when the disclosure is negative.

Sample size

For each oRibo-PACE experiment, up to 4 lagoons were simultaneously used and typically 8 variants per lagoon werr characterized at the end of the experiment. For validation assays using cellular reporters, sample size was based on a 96-well plate grid, with functional assays run with a minimum of 4 biological replicates in all cases.

Data exclusions

No data was excluded.

Replication

For functional characterization of o-ribosome phenotype: sfGFP expression experiments were carried out with 4 (or more) unique colonies from a single transformation on the same day. LuxAB expression assays were limited in throughput by the dimensions of a 96-well plate, so characterization experiments were run over multiple days with the same control run on each day. O-ribosome biological replicates correspond to individual colonies from a single transformation. Doubling time analysis was limited by the dimensions of a 96-well plate and was run over multiple days with the same control being run each day. AHA incorporation assays were run over multiple days with the same controls run on each day.

Randomization

This study utilized clonal E. coli, so randomization was not performed.

Blinding

Blinding was not performed in this study.

Reporting for specific materials, systems and methods

We require information from authors about some types of materials, experimental systems and methods used in many studies. Here, indicate whether each material, system or method listed is relevant to your study. If you are not sure if a list item applies to your research, read the appropriate section before selecting a response.

Materials & experimental systems

n/a	Involved in the study
☒	☐ Antibodies
☒	☐ Eukaryotic cell lines
☒	☐ Palaeontology and archaeology
☒	☐ Animals and other organisms
☒	☐ Human research participants
☒	☐ Clinical data
☒	☐ Dual use research of concern

Methods

n/a	Involved in the study
☒	☐ ChIP-seq
☐	☒ Flow cytometry
☒	☐ MRI-based neuroimaging

Flow Cytometry

Plots

Confirm that:

☒ The axis labels state the marker and fluorochrome used (e.g. CD4-FITC).

☒ The axis scales are clearly visible. Include numbers along axes only for bottom left plot of group (a 'group' is an analysis of identical markers).

☒ All plots are contour plots with outliers or pseudocolor plots.

☒ A numerical value for number of cells or percentage (with statistics) is provided.

Methodology

Sample preparation

For AHA and vitality labeling, SQ171 strains were grown in M9 minimal media (without methionine). Cells were labeled in culture with 200 µM L-azidohomoalanine (AHA) (Click Chemistry Tools) and BacLight Redox Sensor Green Vitality Kit (Invitrogen). Reactions were stopped by adding 200 µg/ mL chloramphenicol and 10 mM NaN_3. Cells were washed in 0.5 mL PBS, fixed with 3.8% PFA, washed twice with PBS, permeabilized with 0.2% Triton X-100 and washed twice more in PBS. Fixed

	and permeabilized cells were mixed with Click-&-Go Reaction Buffer (Click Chemistry Tools) containing 2.5 µM AlexaFlour 405 Alkyne (Click Chemistry Tools) according to manufacturer's instruction and washed twice with PBS prior to sample analysis.
Instrument	Labelled cells were analyzed with BD Biosciences flow cytometer LSR II HTS with excitation lasers at 405, 488 and 561 nm and emission filters at 450/50, 515/20 and 610/20 nm.
Software	Data was analyzed with (FlowJo v10).
Cell population abundance	For protein synthesis and vitality labeling experiments a minimum of 2500 labeled cells were analyzed.
Gating strategy	FSC and SSC gating were used to exclude particles smaller than a bacterial cell. PBS and media without cells was analyzed in order to define background particle sizes for the FSC and SSC gating. Cells treated with 10 mM NaN3 or 200 µg/ mL Chloramphenicol were used as negative controls for vitality staining and AHA labeling, respectively, and these negative controls were used to validate that events in the FSC and SSC gating represented live cells.

☒ Tick this box to confirm that a figure exemplifying the gating strategy is provided in the Supplementary Information.
